# *Gpr75* Deletion in Adipocytes Protects From Diet‐Induced Obesity: Changes in Glucose Homeostasis and Inflammatory Responses

**DOI:** 10.1096/fj.202504597R

**Published:** 2026-02-18

**Authors:** Sakib Hossain, Elizabeth Villegas, Catherine Liapes, Nahid Sultana, Dorina Krasniqi, Danielle Diegisser, Ana Castro, Comfort Williams, Artiom Gruzdev, Darryl C. Zeldin, Victor Garcia, Michal Laniado Schwartzman

**Affiliations:** ^1^ Department of Pharmacology, School of Medicine, New York Medical College Valhalla New York USA; ^2^ National Institute of Environmental Health Sciences, National Institutes of Health North Carolina USA

**Keywords:** diabetes, GLP‐1, GPR75, insulin sensitivity, obesity, sex differences

## Abstract

Loss of function G‐protein coupled receptor 75 (GPR75*)* variants in humans are associated with leanness, and *Gpr75* null mice are protected from diet‐induced obesity (DIO). However, the mechanisms underlying this protection are largely unknown. Here, we investigated the contribution of adipocyte‐derived *Gpr75* to DIO. Adipocyte‐specific *Gpr75* knockout (adipo‐*Gpr75* ^−/−^) male and female mice and their wild‐type (WT) littermates were placed on a high‐fat diet (HFD) for 14 weeks. Metabolic parameters including body weight, energy intake and expenditure, activity, and glucose metabolism were monitored before and after diet feeding. While WT mice obtained a diabetogenic phenotype on HFD, the adipo‐*Gpr75* ^−/−^ counterparts were protected. This protection showed sexual dimorphism. Female adipo‐*Gpr75* ^−/−^ mice displayed a 50% (*p* < 0.001) decrease in weight gain and adiposity compared to WT, whereas male adipo‐*Gpr75* ^−/−^ gained weight like WT mice. Interestingly, both male and female adipo‐*Gpr75* ^−/−^ mice exhibited improved glucose handling compared to WT, which was correlated to decreased adiposity, abrogated adipose tissue inflammation, and increased insulin sensitivity in skeletal muscle. Importantly, no differences in food intake were observed; however, adipo‐*Gpr75* ^−/−^ mice exhibited increased activity and energy expenditure, regardless of sex. Taken together, these findings demonstrate that deletion of GPR75 specifically in adipocytes is sufficient to confer protection against DIO and suggest that adipocyte‐derived GPR75 contributes importantly to the pathogenesis of DIO potentially by mechanisms that may include promotion of inflammation, impairment of insulin signaling, and disruption of metabolic homeostasis.

## Graphical abstract

Diet‐driven weight gain exhibited sex differences; weight gain of adipocyte‐deficient Gpr75 (adipo‐Gpr75^−/−^) female mice was greatly attenuated compared to wild‐type (adipo‐Gpr75^+/+^) mice, whereas male adipo Gpr75^−/−^ mice gained weight similar to wild type. Nevertheless, compared to wild‐type adipo‐Gpr75^+/+^ mice, which showed all the characteristics of diet‐induced obesity, both male and female adipo‐Gpr75^−/−^ mice were protected from diet‐induced metabolic complication exemplified by attenuated adiposity and adipose tissue inflammation, increased activity, and healthy glucose homeostasis.

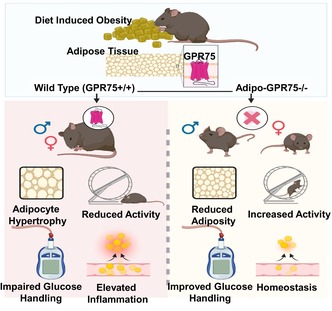

## 1 Introduction

Obesity (BMI ≥ 30) is linked to several prevalent and costly medical conditions including type 2 diabetes (T2D), hypertension, coronary heart disease, stroke and liver disease as well as increased disability and mortality. Obesity prevalence has reached alarming levels. As of 2022, about 16% of adults are obese, and the rate of overweight and obesity combined is approximately 43% of the global adult population [1]. In the United States, 42.4% of adults and 20% of children are classified as obese. The rate of overweight and obesity combined amounts to 73.6% of the US adult population. By 2030, obesity is expected to increase to over 50% of the US population and more than 20% globally [2]. The rapid rise in obesity rates calls for innovative prevention and management strategies to combat this major health crisis with far‐reaching consequences for public health.

Obesity is a complex and highly polygenic condition influenced by numerous genes that regulate food intake and energy expenditure, as well as their interactions with environmental and behavioral factors. Genetic factors are estimated to account for up to 40% of the variation in body mass index (BMI). Recently, we have added to the long list of obesity genes the orphan G‐protein coupled receptor (GPCR) 75 (GPR75). This addition is based on a seminal study indicating that loss of function GPR75 variants in humans are associated with leanness and protection from obesity and that GPR75 null (Gpr75^−/−^) mice are protected from HFD‐driven adiposity and insulin resistance [3]. Additionally, decreased GPR75 expression is associated with lower body weight in humans [4, 5] and protection from diet‐induced obesity in mice and rats [6, 7, 8, 9, 10].

The mechanisms underlying GPR75's contribution to obesity and associated pathologies are largely unknown [11, 12, 13, 14]. Studies in *Gpr75* ^*−/−*^ mice suggested that the mechanisms underlying protection from obesity may include regulation of metabolism in both adipose tissue and brain [7, 9]. To understand how deleting or blocking Gpr75 protects against obesity, it is necessary to create conditional mouse models targeting tissues closely involved in diet‐induced obesity and related cardiometabolic diseases. In this study, we chose to genetically disrupt GPR75 expression in adipose tissue since it is an organ central to the pathogenesis of obesity. Adipose tissue dysfunction in obesity, that is, the inability to buffer excessive caloric intake by increasing adipose tissue mass (adipocyte hyperplasia) leading to adipocyte hypertrophy, is a key factor in the pathophysiology of obesity‐related chronic metabolic diseases. Moreover, adipose tissue expresses *Gpr75* which tend to increase in response to HFD [7].

The present study explores the consequences of adipocyte‐specific genetic disruption of *Gpr75* on diet‐driven obesity and provides some insights into the mechanisms underlying the protection against metabolic complications in response to diet.

## 2 Materials and Methods

### 2.1 Generation of Adipocyte‐Specific *Gpr75* Knockout Mice

The conditional null (“Flox”) *Gpr75* locus was generated by CRISPR/Cas9‐mediated targeting in G4 (B6129F1) embryonic stem (ES) cells as described [15]. Briefly, the entire canonical GPR75 coding sequence, which is entirely in exon 2, was flanked by *LoxP* sites, and following Cre‐mediated recombination, the entire *Gpr75* coding sequence is excised. The primary *Gpr75* flox allele was maintained as a perpetual backcross to wild‐type C57BL/6 J mice (Jackson Laboratory, Bar Harbor, ME, USA). To generate adipo‐*Gpr75* knockout (adipo‐*Gpr75* ^−/−^), we crossed the *Gpr75* flox mice with non‐inducible Adipoq‐Cre (B6.FVB‐Tg(Adipoq‐cre)1Evdr/J, The Jackson Laboratory, Stock #028020) mice. Genotyping of the flox and null mouse colonies was done by Transnetyx (Cordova, TN, USA) using allele‐specific primer/probe assays. Validation of adipocyte‐specific deletion was done by RT‐PCR on freshly isolated adipocytes and adipose tissues taken from adipo‐*Gpr75* ^−/−^ mice and littermate wild‐type adipo‐*Gpr75* ^+/+^ (WT) mice. Adipocytes were isolated from white adipose tissues and separated from the stromal‐vascular fraction (SVF) following published methods [16]. Since there are no reliable antibodies against the mouse GPR75, validation of adipocyte targeting was done by RT‐PCR and RNAscope.

### 2.2 Animal Experimentation

All experimental protocols were approved by the Institutional Animal Care and Use Committee in accordance with the National Institutes of Health Guidelines for the Care and Use of Laboratory Animals. Male and female WT and adipo‐*Gpr75* ^−/−^ mice (10–12‐week‐old) were housed in static polycarbonate caging units (29.2 × 19.1 × 12.7 cm shoebox style) with stainless‐steel wire bar lids, and 7‐cm‐deep isolator cage filter tops in a pathogen‐free facility. Three to four animals were placed in a cage with free access to food (regular chow, LabDiet 5001, St. Louis, MO, USA) and acidified water, 12‐h/12‐h light/dark cycle (LDC), and a temperature between 21°C and 24°C with 30%–70% humidity. The welfare of all animals was monitored daily by licensed veterinary technicians employed by New York Medical College. Mice were placed on an HFD (cat. no. 03584; Envigo, Huntingdon, UK). The composition of the HFD diet in percent kilocalories (kcal) was 58.4% fat, 26.6% carbohydrate, and 15.0% protein. All animals were monitored for changes in body weight on a weekly basis. Body composition (Micro‐CT), fasting blood glucose, and glucose tolerance test were measured before and at week 14. Assessment of energy intake and expenditure, as well as activity and respiratory exchange ratio, was done before and after 14 weeks of HFD feeding and over a period of 5–7 days using the Promethion Sable Systems metabolic cages (North Las Vegas, NV, USA). At the end of the experiment, mice were anesthetized with intraperitoneal injection of ketamine (100 mg/kg) and xylazine (10 mg/kg). Blood was withdrawn, and tissues including visceral (VAT), subcutaneous (SAT), and brown (BAT) adipose tissues, as well as skeletal muscle, were harvested for various assays. All monitoring tests and post‐feeding analyses are described below and were done in a blinded fashion.

### 2.3 Assessment of Energy Intake and Expenditure

Mice were housed in metabolic cages (1 per cage) (Promethion, Sable Systems) for 1 week, including acclimation time, at baseline and at the fourteenth week of HFD feeding. The Promethion system contains in‐cage balances that continuously measure food intake and body mass, as well as high‐precision sensors to assess live oxygen consumption (*V*_02_), carbon dioxide production (*V*_CO2_), and activity. Daily monitoring of the system was tracked via Promethion Live. Mice were housed in thermoneutral and light‐controlled conditions (30°C, 12‐h light/12‐h dark cycle from 7:00 am to 7:00 pm), with free access to the food intake module, water intake module, and body weight monitor. Bedding was not changed throughout the week to minimize environmental disruption. Locomotor activity was assessed using pedometers, which measured distance traveled (meters) by walking or running. Energy expenditure was calculated using the equation, $\mathrm{EE}\;\left(\text{kCal}\right)=\left(\left(1.232\times \mathrm{rq}\right)\right)+3.815)\times V_{\mathrm{CO}2}$ and the respiratory exchange ratio (RER) was determined as the ratio of carbon dioxide production to oxygen consumption per mouse. ($\mathrm{RER}=V_{\mathrm{CO}2}/V_{O2}$). To determine whether total body mass (TBM) or lean body mass (LBM) influenced the differences in EEKcal.d between genotypes after HFD feeding, ANCOVA analysis was conducted with grouping variable set to genotype, EE variable set to EEKkcal.d and CoVariate variable set to TBM or LBM.

### 2.4 Micro‐Computed Tomography (mCT) Scanning and Analysis

Whole body mCT scans were obtained from mice anesthetized with isoflurane (4% for induction and 2% during scan acquisition) using the Quantum GX2 mCT imaging system (PerkinElmer, Shelton, CT, USA). Settings were optimized for body composition as follows: a voltage of 70 kV, field of view (FOV) of 72 mm, and an X‐ray filter of Aluminum 1.0 mm. CT scans were acquired for 6 min and analyzed using Analyze 14.0 software (AnalyzeDirect). Fat was isolated for analysis by density threshold. Separation of visceral (VAT) and subcutaneous (SAT) adipose tissue was done by sequential manual tracing of coronal slices in 2D, every 7 slices for the length of the region of interest. Regions of similar density to fat were excluded using the same method to remove air within the lungs and equipment artifacts. The 3D sections are then reconstructed and processed to quantify volume. The fat‐free volume was calculated by subtracting total fat volume from total body volume (total body volume – (visceral fat volume + subcutaneous fat volume)).

### 2.5 Fasting Blood Glucose, GTT, and Assessment of Insulin Resistance

Intraperitoneal glucose tolerance tests (GTT) were performed prior to HFD feeding and at the end of the 14‐week experimental diet. All experiments were conducted promptly at 10:00 a.m. Intraperitoneal injections were chosen for the route of administration to avoid errors that may occur with the oral route of administration. For GTT, after a 12‐h overnight fasting period in clean cages containing Alpha‐dri paper pulp cellulose animal bedding, 20% (vol/vol) dextrose was given through an intraperitoneal injection in a 2 g/kg body weight proportion. Blood glucose levels were measured by tail pinch immediately before and at 15, 30, 60, 90, and 120 min after injection using a Bayer Contour blood glucose monitoring system (7097c). After these measurements, blood was collected in capillary tubes and used for insulin measurements. Blood was centrifuged at 2000 rpm for 15 min to separate the plasma. The Ultra‐Sensitive Mouse Insulin ELISA kit (cat. no. 90080, Crystal Chem, Elk Grove Village, IL, USA) was used to quantify plasma insulin levels as per the manufacturer's instructions. Homeostatic model assessment of insulin resistance (HOMA‐IR) was calculated as follows: fasting blood glucose (mmol/L) × fasting blood insulin (pmol/L)/22.5.

### 2.6 Plasma Adipokines and Cytokines

Bio‐Plex Pro mouse diabetes immunoassay kits (BIO‐RAD, Hercules, CA, USA) were used to measure multiple markers of mouse diabetes and obesity per the manufacturer's protocol. Briefly, magnetic capture antibody‐coupled beads were washed and incubated with diluted (1:4) standards, blanks, and plasma samples in duplicate wells on a black 96‐well microplate. This was followed by the sequential addition of detection antibodies and Streptavidin‐PE for measurement. Data from the reactions were acquired using the Luminex‐200 system (Austin, TX, USA), and results were analyzed with the xPONENT software. Adiponectin was measured using the Bio‐Plex Pro Mouse Diabetes Adiponectin Assay (BIO‐RAD #171F7002M). Levels of adiponectin were used to calculate the leptin to adiponectin ratio, an index of insulin sensitivity. Plasma cytokines were quantified via the Luminex ProcartaPlex Immunoassay Kit (Thermo‐Fisher, Waltham, MA, USA). This assay followed a comparable methodology, where magnetic capture antibody‐coupled beads were washed and incubated with 1:1000 diluted standards, samples, and blanks in duplicate wells on a magnetic 96‐well plate. Detection antibodies and Streptavidin‐PE incubations were done successively. The Luminex‐200 instrument was used for data acquisition, and raw data were exported as a csv file and analyzed using ProcartaPlex Analyst software (Thermo‐Fisher).

### 2.7 Histology

H&E staining was performed using published protocols [7]. High‐resolution whole slides of H&E‐stained adipose were viewed under 20× magnification using an Echo Revolve microscope (Echo, San Diego, CA, USA) in the phase‐contrast setting. Pictures (2732 μm × 1960 μm) were taken of 5 distinct areas in each slide. A free‐hand drawing tool was used to manually trace all cells, and the area of each adipocyte was calculated using ImageJ Software. Scale bar length was manually input into the software to ensure the area of traced objects was aligned to scale.

### 2.8 Immunofluorescence

Mice were sacrificed and their visceral adipose tissue was harvested, fixed in formalin and embedded into paraffin wax. These tissues were then sectioned (5 μm thick), embedded in paraffin, and mounted onto slides. The slides were deparaffinized/rehydrated and microwaved in 1× citrate buffer for antigen retrieval. After washing the slides with deionized water, tissue sections on the slide were blocked with a solution containing 5% normal goat serum in 1× PBS for 1 h at room temperature. The blocking solution was then replaced with primary antibodies including rabbit anti‐CD68 (1:300, Cell signaling; Cat#97778; Danvers, MA, USA) and rat anti‐TREM2 (1:100, Sigma Aldrich; Cat# MABN755, St. Louis, MO, USA), diluted in the blocking solution, and incubated overnight at 4°C. The secondary antibodies were from Thermo‐Fisher (Waltham, MA, USA) and were used at a dilution of 1:500 for goat Anti‐rabbit IgG (H + L), F(ab’)_2_ Fragment (Alexa Fluor 594 Conjugate) and 1:500 for Donkey anti‐Rat IgG (H + L) Highly Cross‐Adsorbed Secondary Antibody (Alexa Fluor 647) in 1× PBS. The slides were incubated with the secondary antibody cocktail for 1 h at room temperature in the dark, followed by DAPI staining (1:7000 dilution in 1× PBS). Once tissue sections were washed with PBS and deionized water, slides were mounted with Fluoromount‐G mounting media. All tissue sections were imaged on the Zeiss LSM 980 plus Airy scan II confocal laser microscope with a 20× objective (Oberkochen, Germany).

### 2.9 qRT‐PCR

Total RNA was extracted from tissue using the miRNeasy Kit (Qiagen, 1038703; Hildan, Germany) according to the manufacturer's protocol. Complementary DNA (cDNA) was then synthesized from total RNA using the QuantiTect Reverse Transcription Kit (Qiagen, 205 311). Quantitative real‐time PCR analysis of cDNA was performed in the QuantStudio 5 Real‐time PCR System using TaqMan Gene Expression Assays from Thermo‐Fisher (*Gpr75*: Mm00558537_s1, Tnf: Mm00443258_m1, *Actb*: Mm02619580_g, *Nfκb1*: Mm00476361_m1, *Adipoq*: Mm04933656_m1) with the following cycling conditions: 50°C for 2 min, 95°C for 10 min, followed by 40 cycles of 95°C for 15 s and 60°C for 1 min. Target gene expression was normalized to that of *Actin*, and quantitative analyses were conducted using the ∆∆cycle threshold method.

### 2.10 RNAscope and Microscopy

All RNAscope reagents were purchased from Advanced Cell Diagnostics (Newark, CA, USA) and are components of the RNAscope Multiplex Fluorescent Reagent kit v2, Catalog # 323100. RNAscope was performed on FFPE slides containing wild‐type and adipocyte‐specific *Gpr75* knockout mouse visceral adipose tissue which were previously and fixed in 10% paraformaldehyde for 24 h before being processed, according to the RNAscope Multiplex Fluorescent Reagent Kit v2 Assay user manual provided by the manufacturer. Slides were baked in a dry oven for 1 HR at 60°C before undergoing deparaffinization processing, which included two 5‐min washes in xylene with slight agitation, then two‐minute washes in 100% ethanol with slight agitation. Slides were dried at room temperature for 5 min. A few drops of RNAscope Hydrogen Peroxide were applied to each tissue section and spread so the tissue was fully covered and was incubated at RT for 10 min. Slides were then washed 2 times in DI water. An Oster Steamer was filled with tap water to the “Max Fill” line, containers filled with DI water and RNAscope 1× Target Retrieval Reagent placed in the steamer at 99°C (checked with scanning thermometer). Slides were acclimated in the DI water for 10 s before being placed in the RNAscope 1× Target Retrieval Reagent for 15 min. Slides were removed and washed with DI water for 15 s before being placed in 100% ethanol for 3 min. After that, slides were dried at room temperature for 5 min. A hydrophobic barrier was drawn around each tissue section and allowed to dry for 1–2 min before applying a few drops of RNAscope Protease Plus to cover each tissue section and allowing to sit in the HybEZ Humidity Control Tray in the HybeEZ Oven at 40°C for 30 min. Slides were then washed in DI water two times. Probes for *Gpr75‐C1* (catalog # 318281) and *Adiponectin‐C2* (catalog # 440051‐C2) were prepared exactly as described by the user manual on the day of the procedure. Tyramide signal amplification (TSA) was used to enhance sensitivity for low‐abundance transcripts. A few drops of the probe mixtures were applied to each tissue section and spread so the entire section was covered before incubating in the HybEZ Humidity Control Tray in the HybeEZ Oven at 40°C for 2 h. Slides were then washed with 1× Wash Buffer and left in 5× SSC Buffer overnight at RT. The following morning the slides were washed twice with 1× Wash Buffer for 2 min at RT. Three Hybridization AMP steps were performed according to the user manual before the HRP C1 (TSA Vivid 650; catalog # 323273) and HRP C2 (TSA Vivid 570; catalog # 323272) signals were developed. Lastly, the slides were mounted and counterstained with DAPI using the SouthernBiotech DAPI Fluoromount‐G (Catalog #: 0100–20) and allowed to dry overnight at RT in the dark. The following day, slides were imaged on a Zeiss LSM 980 + Airyscan 2 High Resolution confocal microscope and images processed using Zen Blue 3.3. Images were taken under 40× (water‐immersed) magnification using three lasers: Cy3 (561 nm at 1.2% strength and 650 V Gain), Cy5.5 (639 nm at 13.0% strength and 760 V Gain), and DAPI (405 nm at 14% strength and 650 V Gain).

### 2.11 Western Blot Analysis

At the termination of the experiment, mice were euthanized, and tissues were snap‐frozen in liquid nitrogen and stored at −80°C. Skeletal muscle was homogenized in RIPA lysis buffer (Milipore Sigma, Burlington, MA, USA) containing protease and phosphatase inhibitors (Roche, Mannheim, Germany). Homogenates were centrifuged, and the lysates were collected and processed for Western blot analysis. Protein extraction from adipose tissues went through acetone precipitation in order to eliminate lipid contamination as described [17]. Protein concentration was determined by Pierce BCA Protein Assay Kit (Thermo‐Fisher Scientific, Waltham, MA, USA). Samples (20 μg of protein) were separated using 4%–20% Mini‐PROTEAN TGX precast gel (Bio‐Rad, Hercules, CA, USA). Proteins in the gel were then transferred to nitrocellulose membrane via Mini Trans‐Blot Cell (Bio‐Rad). Blots were blocked with 5% BSA blocking buffer for 1 h at room temperature, incubated with primary antibody (1:1000 dilution) overnight at 4°C, followed by incubation with goat anti‐rabbit or goat anti‐mouse IgG antibody (1:10 000 dilution) conjugated with IR Dye 800CW (LI‐COR Biosciences, Lincoln, NE, USA) at room temperature for 1 h. Membranes were subsequently washed with 1X TBST, visualized and quantified with lane normalization factor of the housekeeping protein using LI‐COR Odessey CLx imaging system and Image studio software version 5.2.5. The following primary antibodies were purchased from Cell Signaling Technologies (Danvers, MA, USA): Akt (cat # 9272), pAkt serine 473 (cat # 9271), Glut 4 (cat # 2213), UCP‐3 (cat # 97000), UCP1 (cat # 14670), and β‐actin (cat # 3700). Antibody against pIR tyrosine 1334 (cat # 44‐809G) was purchased from Thermo‐Fisher Scientific (Waltham, MA, USA).

### 2.12 Statistical Analysis

A power analysis was performed using Power & Precision 4 software. Assuming there is > 50% difference in the means between the groups with 95% power and α = 0.05, 8 male and 8 female mice/group were required to reach significance. Statistical comparisons were performed using an unpaired *t*‐test and 2‐way ANOVA followed by a post hoc Tukey's test (Graph Pad Prism version 9.2.0 software). The following strategy was taken: (a) WT and Gpr75 transgenic mice randomly distributed in different experimental groups; (b) all the studies were performed in a blinded fashion; and (c) each in vitro assay was conducted in duplicate with appropriate *n*‐values.

## 3 Results

### 3.1 Validation of *Gpr75* Knockdown in Adipocytes

**FIGURE 1:**
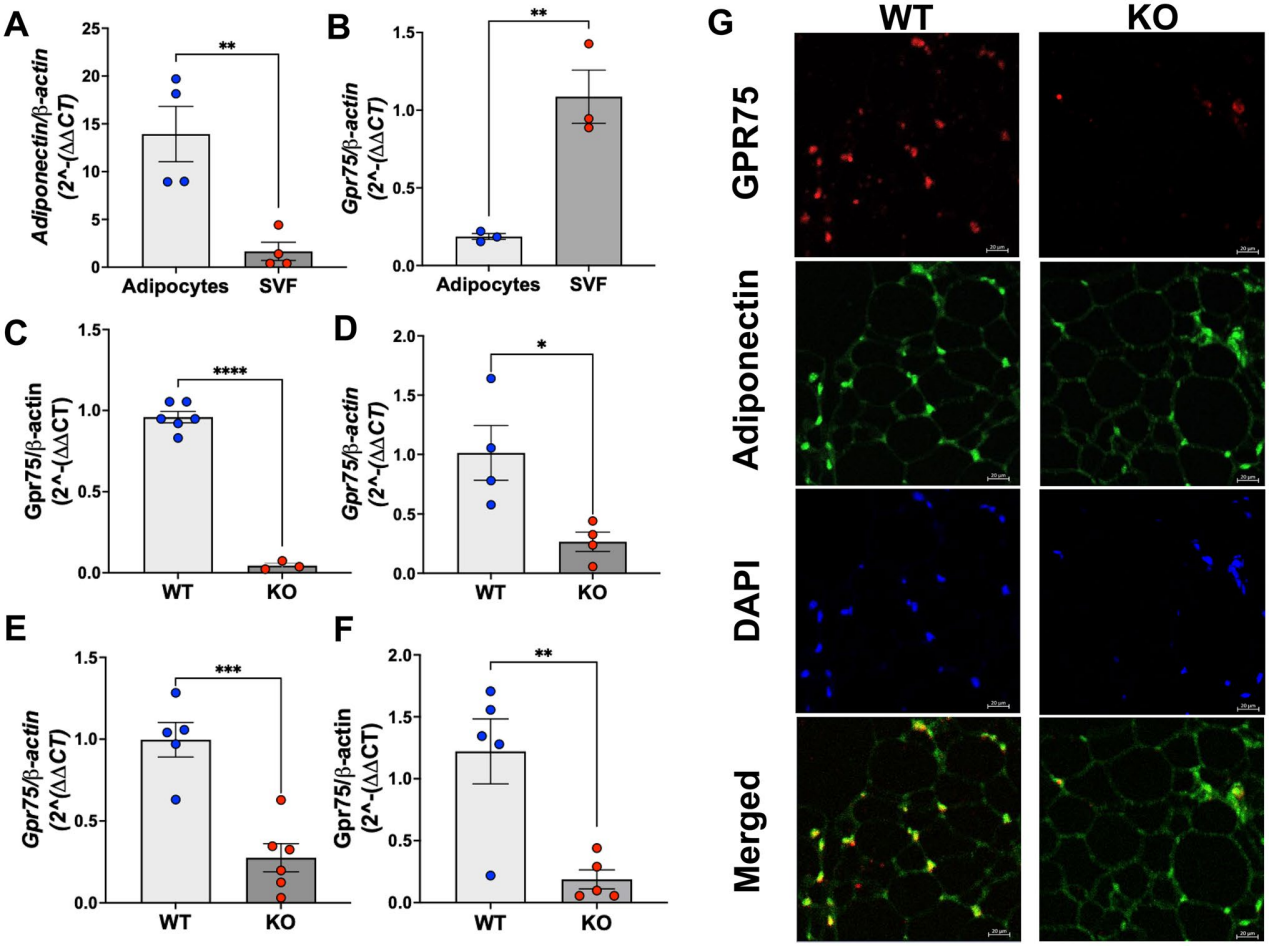
Validation of adipocyte targeting. (A) Isolation of adipocytes from adipose tissues was confirmed by measuring adiponectin expression in the adipocyte fraction and the stromal‐vascular fraction (SVF), (B) *Gpr75* mRNA levels in adipocyte and SVF from adipo‐*Gpr75* ^−/−^ (KO) mice. (C) levels of *Gpr75* mRNA in isolated adipocytes from WT and adipo‐*Gpr75* ^−/−^ (KO) mice. *Gpr75* mRNA levels in brown adipose tissue (BAT) from female (D) and male (E) WT and adipo‐*Gpr75* ^−/−^ (KO) mice, and (F) in subcutaneous adipose tissue (SAT) from male WT and adipo‐*Gpr75* ^−/−^ (KO) mice. (G) RNAscope of *Gpr75* and *Adiponectin* in visceral fat from male WT and adipo‐*Gpr75* ^−/−^ (KO) mice. Images were taken at 40× using the confocal microscope as described in methods; the bar depicted in each image is 20 μm. Statistical significance between two group was determined by unpaired t‐test with Welch's corrections (ns, not significant; *p < 0.05, **p < 0.01, ***p < 0.001 and ****p < 0.0001).

Adipocytes were isolated from the gonadal adipose tissues of WT and adipo‐*Gpr75* ^−/−^ mice. Results of validation are presented in Figure 1. Assessment of the adipocyte‐specific marker adiponectin showed an 8‐fold higher expression compared to the stromal‐vascular fraction (SVF), indicating the enrichment of adipocytes during the process of isolation (Figure 1A). While the entire GPR75 coding sequence is excised in our global *Gpr75* null mice, all commercial and custom GPR75 antibodies have non‐specific staining; therefore, confirmation of tissue‐specific disruption must be done either at the transcript or genomic level. We employed the use of RT‐PCR and RNAscope to evaluate its gene expression level. Levels of *Gpr75* mRNA in the adipocyte fraction from adipo‐*Gpr75* ^−/−^ mice were 5‐fold lower than in the corresponding SVF fraction (Figure 1B). Importantly, expression of *Gpr75* mRNA in adipocytes from adipo‐*Gpr75* ^−/−^ mice was 9‐fold lower than in WT adipocytes (Figure 1C). These results strongly indicated an effective adipocyte‐selective knockdown of *Gpr75* in the adipo‐*Gpr75* ^−/−^ mice. *Gpr75* deficiency was also evident in adipose tissues of adipo‐*Gpr75* ^−/−^ mice. For example, *Gpr75* expression was about 75% lower in the brown adipose tissue (BAT) of male and female adipo‐*Gpr75* ^−/−^ mice (Figure 1D,E) and nearly 85% lower in the subcutaneous adipose tissues (SAT) of male adipo‐*Gpr75* ^−/−^ mice compared to WT (Figure 1F). RNAscope of adipose tissue from adipo‐*Gpr75* ^−/−^ mice demonstrated a lack of GPR75 expression within the adipocytes (Figure 1G). Notably, Gpr75 mRNA levels in brain and liver tissues were not different between WT and adipo‐*Gpr75* ^−/−^ mice (Figure S1). For all genotypes in this study, no naïve phenotype was observed that impacted viability, mendelian breeding, nor gross morphological analysis. Multiple complementary approaches, including routine genotyping, longitudinal phenotypic monitoring, and reproducible molecular signatures observed across independent cohorts, reinforced further the overall rigor and reproducibility of the animal model (vide infra).

### 3.2 Body Weight Increases in Response to HFD in Adipocyte‐Specific *Gpr75*‐Deficient Mice

**FIGURE 2:**
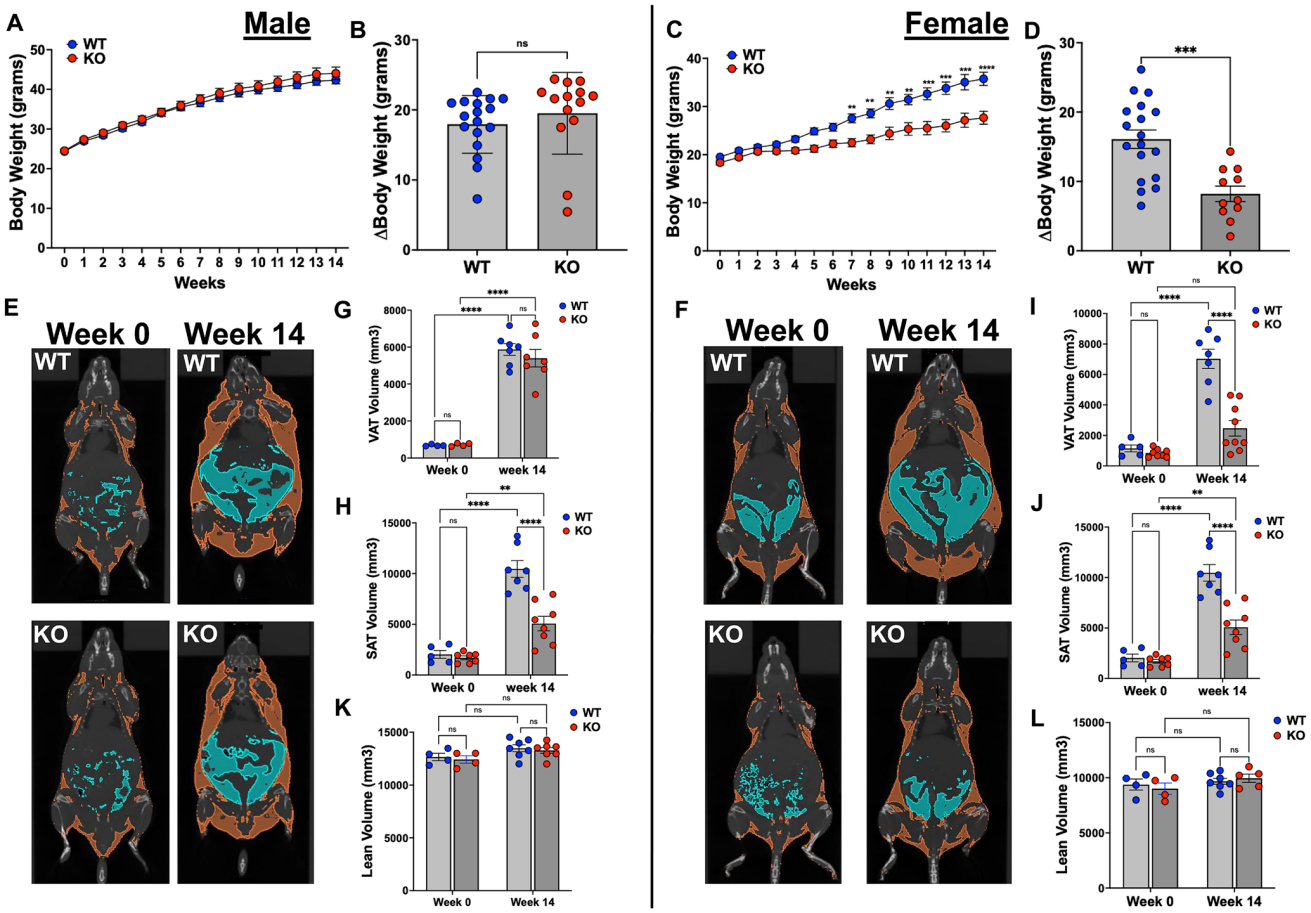
Body weight and adiposity in 14‐week‐HFD‐fed male and female WT and adipo‐*Gpr75* ^−/−^ mice. Body weight was averaged weekly. Micro‐CT was performed before and after 14 weeks of HFD feeding and fat as well as lean volume calculated. Body weight trajectories and weight gain in male (A, B) and female (C, D) WT and adipo‐*Gpr75* ^−/−^ (KO) mice. Representative Micro‐CT images of male (E) and female (F) WT and adipo‐*Gpr75* ^−/−^ (KO) mice before and after 14 weeks of HFD feeding. Volume of visceral (VAT in blue) and subcutaneous (SAT in orange) adipose tissues in male (G, H) and female (I, J) WT and adipo‐*Gpr75* ^−/−^ (KO) mice before and after 14 weeks of HFD feeding. Lean volume of male (K) and female (L) WT and adipo‐*Gpr75* ^−/−^ (KO) mice before and after 14 weeks of HFD feeding. Results are mean ± SE. Statistical significance between two group was determined by unpaired t‐test with Welch's corrections and between multiple groups by two‐way ANOVA with Tukey's multiple comparison (ns, not significant; **p < 0.01, ***p < 0.001, and ****p < 0.0001).

The body weights (mean ± SEM) of WT and adipo‐*Gpr75* ^−/−^ mice at the onset of HFD feeding were similar and amounted to 24.37 ± 0.60 and 24.51 ± 0.43 g for male WT and adipo‐*Gpr75* ^−/−^ mice, respectively, and 19.28 ± 0.45 and 18.34 ± 0.49 g for female WT and adipo‐*Gpr75* ^−/−^ mice, respectively. As seen in Figure 2, HFD feeding over 14 weeks gradually increases body weight in both males and females. While body weight gain in response to HFD feeding was similar for male WT and adipo‐*Gpr75* ^−/−^ (Figure 2A,B), female adipo‐*Gpr75* ^−/−^ only gained 51% compared to their WT counterpart (8.22 ± 1.11 vs. 16.09 ± 1.33 g, body weight change for female adipo‐*Gpr75* ^−/−^ and WT mice, respectively) (Figure 2C,D). Notably, the attenuated weight gain displayed by adipocyte‐specific adipo‐*Gpr75* ^−/−^ female mice is comparable to that observed in *Gpr75* ^*+/−*^ female mice [7] (51% and 50%, respectively).

### 3.3 Adipocyte *Gpr75* Deficiency Reduces Adiposity, Adipocyte Hypertrophy, and Adipose Inflammation in Response to HFD

Assessment of adipose tissue volumes by micro‐CT closely paralleled body weight gain of male (Figure 2E) and female (Figure 2F) WT and adipo‐*Gpr75* ^−/−^ mice. In males, VAT and SAT volumes increased in response to HFD feeding by 6‐ and 10‐fold, respectively, in both WT and adipo‐*Gpr75* ^−/−^ mice (Figure 2G,H). In contrast, the HFD‐driven increases in adipose tissue volumes were significantly attenuated in female adipo‐*Gpr75* ^−/−^ mice as compared to corresponding WT; the volume of VAT increased by 7‐fold in female WT, but it was only increased by approximately 2‐fold in female adipo‐*Gpr75* ^−/−^ (Figure 2I). Similarly, the increase in SAT volume was largely attenuated in female adipo‐*Gpr75* ^−/−^ compared to WT (Figure 2J). Noteworthy is the finding that there were no significant changes in lean mass in response to HFD feeding in both male and female WT and adipo‐*Gpr75* ^−/−^ mice (Figure 2K,L).

**FIGURE 3:**
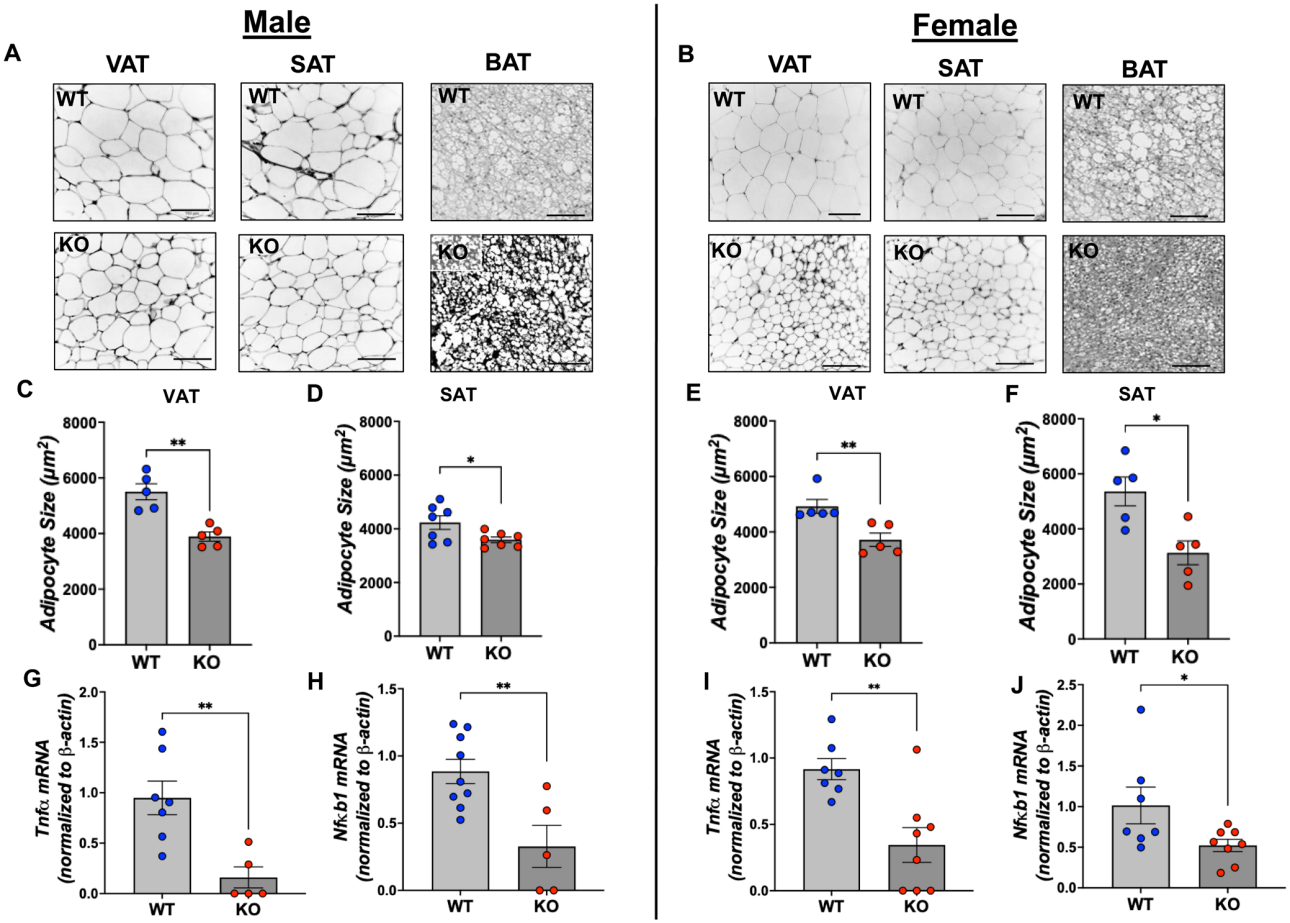
Adipose tissue morphology and inflammatory markers in 14‐week‐HFD‐fed male and female WT and adipo‐*Gpr75* ^−/−^ mice. Representative images of H&E staining in visceral (VAT), subcutaneous (SAT) and brown (BAT) adipose tissues of male (A) and female (B) WT and adipo‐*Gpr75* ^−/−^ (KO) mice. All images were taken at 20× magnification with scale bar represent 110 μm. Adipocyte size, in VAT and SAT of male (C, D) and female (G, H) WT and adipo‐*Gpr75* ^−/−^ (KO) mice. Expression of *Tnfα* and *Nfκb1* mRNA in SAT from male (E, F), and female (I, J) WT and adipo‐*Gpr75* ^−/−^ (KO) mice. Results are mean ± SE; ns, not significant; *p < 0.05, and **p < 0.01 by unpaired *t*‐test with Welch's corrections.

Adipose tissue histology indicated that genetic disruption of Gpr75 in adipose tissue attenuated HFD‐driven adipocyte hypertrophy. At the onset of HFD feeding, adipocyte size in VAT and SAT was not different among the genotypes (Figure S2). HFD feeding resulted in adipocyte hypertrophy; however, it was significantly attenuated in both male and female adipo‐*Gpr75* ^−/−^ mice. Hence, adipocyte size of VAT (*p* < 0.01) and SAT (*p* < 0.05) from male adipo‐*Gpr75* ^−/−^ mice at the end of HFD feeding was lower compared to WT mice (Figure 3A,C,D). Likewise, in female mice, adipocyte size in both VAT (25%) and SAT (45%) was significantly lower in adipo‐*Gpr75* ^−/−^ compared to WT mice (Figure 3B,E,F). The density of adipocytes within brown adipose tissue (BAT) and their small size made it difficult to assess and determine differences between experimental groups. Importantly, expression of the inflammatory markers *Tnfα* and *Nfkb1* in adipose tissues from either male (Figure 3G,H) or female (Figure 3I,J) HFD‐fed adipo‐*Gpr75* ^−/−^ mice was 50%–90% lower than in corresponding WT mice. The reduction in adipose inflammatory markers was associated with lower circulatory levels of several inflammatory cytokines in female adipo‐*Gpr75* ^−/−^ mice, including TNFα, IL‐1, IL‐2, MCP‐1, and KC. On the other hand, in male adipo‐*Gpr75* ^−/−^, there were increases in levels of several inflammatory chemokines and cytokines including MCP‐1 (2.5‐fold), KC (2.3‐fold), IL‐1β (6.3‐fold), and IL‐2 (3.8‐fold) compared to WT mice (Figure S3).

Notably, levels of RANTES (CCL5), a putative GPR75 ligand/interactor [18], were unchanged in both male and female adipo‐*Gpr75* ^−/−^ mice compared to WT mice.

**FIGURE 4:**
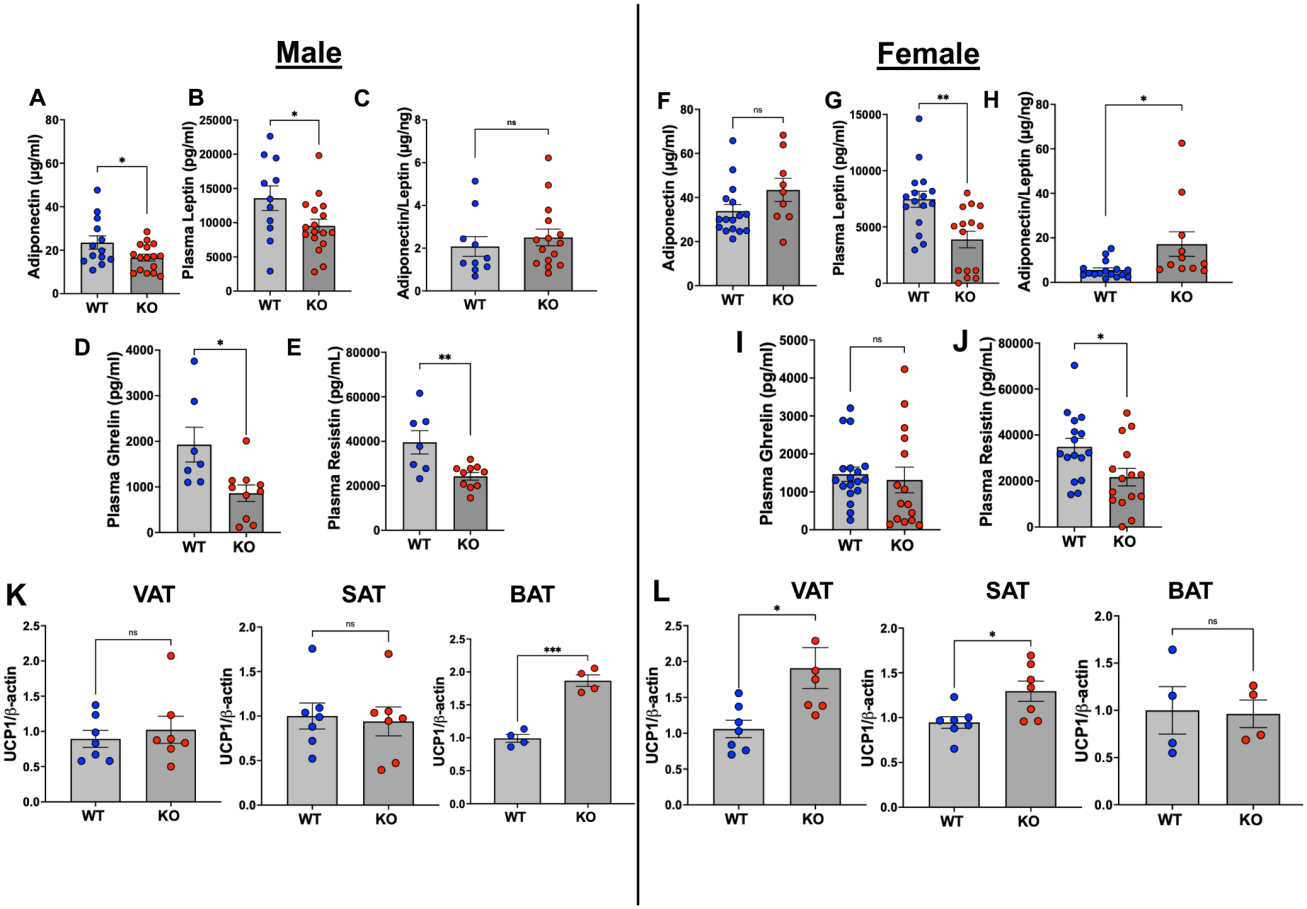
Levels of Circulatory adipokines and adipose tissue UCP1 in 14‐week‐HFD‐fed male and female WT and adipo‐*Gpr75* ^−/−^ mice. Plasma levels of Adiponectin (A, F), Leptin (B, G), adiponectin to leptin ratio (C, H), Gherlin (D, I) and resistin (E, J) in male and female, respectively, WT and adipo‐*Gpr75* ^−/−^ (KO) mice. Densitometry analysis of Western blots of UCP1 relative to β‐actin in visceral (VAT), subcutaneous (SAT), and Brown (BAT) from male (K) and female (L) WT and adipo‐*Gpr75* ^−/−^ (KO) mice. Results are mean ± SE; ns, not significant; *p < 0.05, **p < 0.01, and ***p < 0.001 by unpaired t‐test with Welch's corrections.

The health of the adipocytes was further reflected by levels of circulatory adipokines. In males, levels of adiponectin and leptin (Figure 4A,B) as well as ghrelin and resistin (Figure 4D,E) were significantly lower in adipo‐*Gpr75* ^−/−^ mice compared to the WT. In females, levels of adiponectin and ghrelin were not different between WT and adipo‐*Gpr75* ^−/−^ mice (Figure 4F,I), while levels of leptin and resistin were significantly lower in adipo‐*Gpr75* ^−/−^ as compared to WT (Figure 4G,J). We also calculated the adiponectin to leptin ratio which serves as a marker for adipose tissue dysfunction and insulin resistance. Conventionally, in mice, this ratio decreases significantly in response to HFD correlating with increased body weight, adiposity, and insulin resistance [19]. In our study, this ratio was significantly higher in female adipo‐*Gpr75* ^−/−^ mice, whereas no significant differences were noted in male mice (Figure 4C,H, respectively). Interestingly, levels of uncoupling protein 1 (UCP1), a marker for thermogenesis whose activation is linked to improved glucose homeostasis, increased insulin sensitivity, and protection against diet‐induced obesity [20], were significantly higher in VAT from male and female adipo‐*Gpr75* ^−/−^ mice compared to WT mice following 14 weeks of HFD feeding. Elevated UCP‐1 protein expression was also noted in BAT of male adipo‐*Gpr75* ^−/−^ and SAT of female adipo‐*Gpr75* ^−/−^ (Figure 4K,L and Figure S4).

**FIGURE 5:**
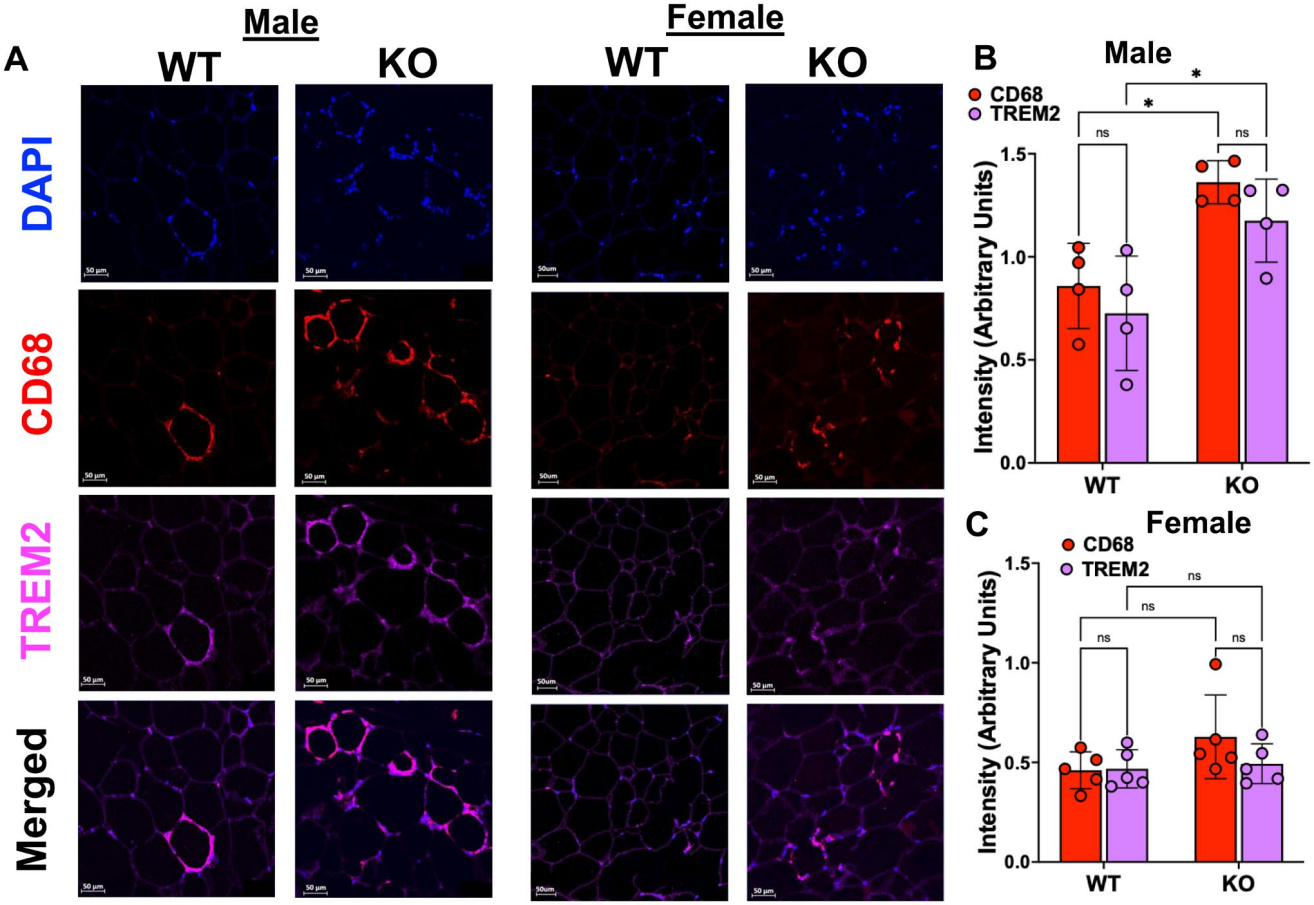
Lipid‐associated macrophages in adipose tissues from 14‐week‐HFD‐fed male and female WT and adipo‐*Gpr75* ^−/−^ mice. (A) Representative immunofluorescence images of VAT sections from HFD‐fed male and female WT and adipo‐*Gpr75* ^−/−^ (KO) mice stained with CD68 (red), TREM2 (purple), DAPI (blue) antibodies. Images were taken with 20× objective, size bar is 50 μm. Estimation of CD68 and TREM2 fluorescence intensity in VAT from male (B) and female (C) WT and adipo‐*Gpr75* ^−/−^ (KO) mice. Results are mean ± SE; ns, not significant; *p < 0.05 by two‐way ANOVA with Tukey's multiple comparison test.

Intriguingly, CD68 immunofluorescence which is seen as crown‐like structures around large adipocytes in VAT sections from HFD‐treated mice was greater in adipo‐*Gpr75* ^−/−^ mice compared to WT, primarily in male mice (Figure 5A). These CD68 positive cells are possibly lipid‐associated macrophages (LAM) which are known to accumulate within adipose tissues in response to excess extracellular lipids driven by HFD. These specialized macrophages function to preserve tissue integrity in the face of adipocyte cell death by promoting anti‐inflammatory lipid clearance [21]. Lipid‐associated macrophages express signature proteins including the triggering receptors expressed on myeloid cells 2 (TREM2), which drives a gene expression program involved in phagocytosis, lipid catabolism, and energy metabolism [21]. Indeed, double staining with CD68 and TREM2 antibodies showed co‐localization indicating the presence of TREM2‐positive LAM, the number of which was significantly higher in male (Figure 5B) but not in female (Figure 5C) adipo‐*Gpr75* ^−/−^ mice. This finding suggests that GPR75 deficiency allowed for the accumulation of TREM2‐positive LAM which contributed to adipocyte metabolic health under the condition of lipid overload. The greater increase seen in adipose tissue from male adipo‐*Gpr75* ^−/−^ mice may be due to the increasing levels of chemokines such as MCP‐1 and KC (Figure S2). At baseline, CD68‐TREM2 positive cells were barely detectable in either WT or adipo‐*Gpr75* ^−/−^ of male or female mice (Figure S5).

### 3.4 Adipocyte *Gpr75* Deficiency Maintains Glucose Homeostasis and Insulin Sensitivity in Response to HFD

**FIGURE 6:**
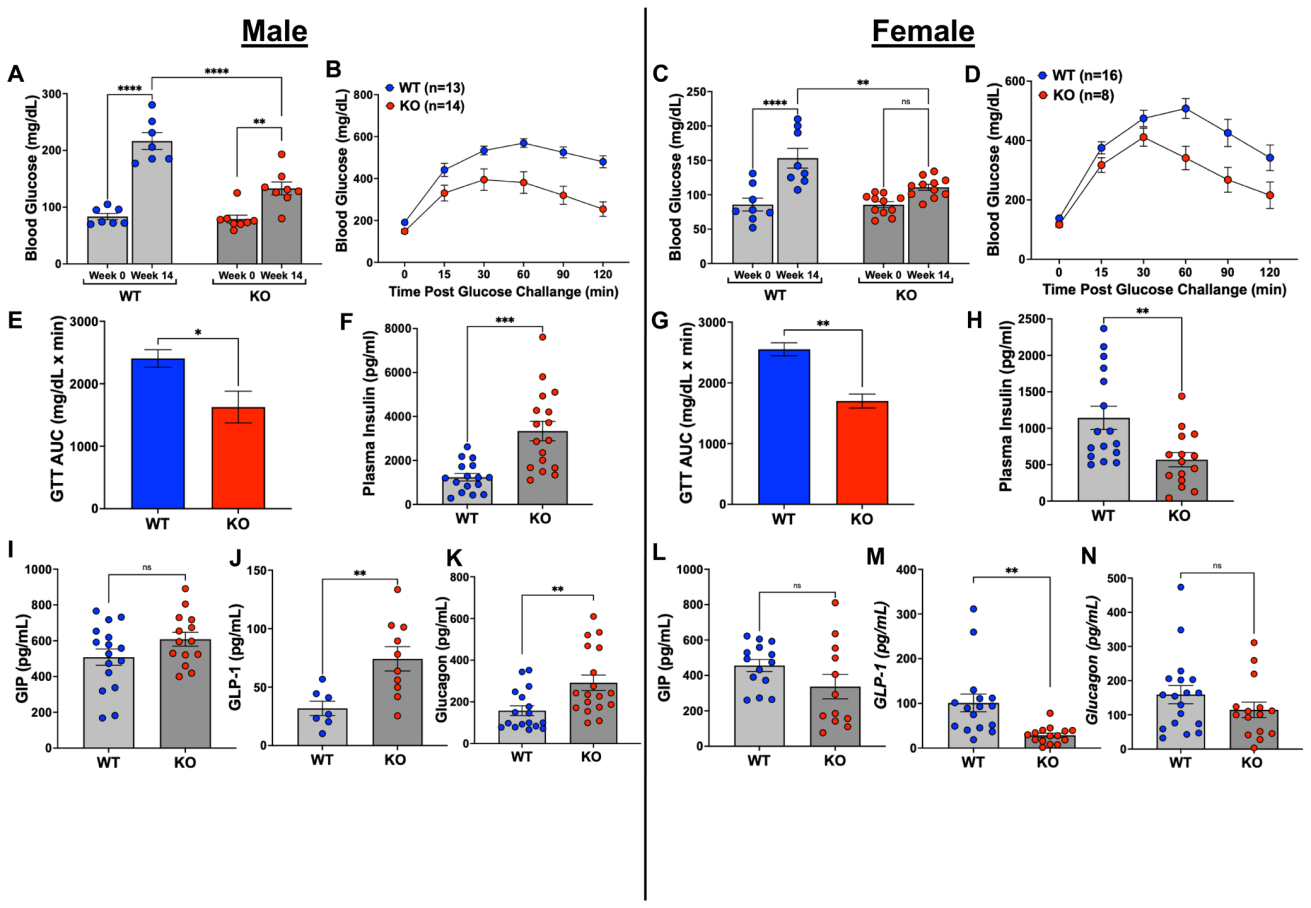
Fasting blood glucose, glucose tolerance test (GTT) and levels of circulatory insulin and incretins in 14‐week‐HFD‐fed male and female WT and adipo‐*Gpr75* ^−/−^ mice. Fasting blood glucose and GTT in male (A, B) and female (C, D) WT and adipo‐*Gpr75* ^−/−^ (KO) mice. GTT area under the curve and fasting plasma insulin in male (E, F) and female (G, H) WT and adipo‐*Gpr75* ^−/−^ (KO) mice after 14 weeks of HFD feeding. Plasma GIP, GLP‐1, and glucagon in male (I–K) and female (L–N) WT and adipo‐*Gpr75* ^−/−^ (KO) mice fed HFD for 14 weeks. Statistical significance between two groups was determined by unpaired *t*‐test Welch's corrections and between multiple groups by two‐way ANOVA with Tukey's multiple comparison (ns, not significant; *p < 0.05, **p < 0.01, ***p < 0.001, and ****p < 0.0001).

HFD‐feeding increased fasting blood sugar (FBS) in both male (Figure 6A) and female (Figure 6C) WT mice. This HFD‐driven increase was greatly attenuated in male adipo‐*Gpr75* ^−/−^ mice (Figure 6A). In female adipo‐*Gpr75* ^−/−^, FBS did not increase in response to HFD (Figure 6C). Intraperitoneal glucose tolerance tests performed at the end of the HFD feeding period showed that both male (Figure 6B) and female (Figure 6D) adipo‐*Gpr75* ^−/−^ mice cleared glucose from the blood significantly better than WT, indicating better glucose handling. Analysis of the area under the curve further showed that male (Figure 6E) and female (Figure 6G) adipo‐*Gpr75* ^−/−^ mice have better glucose tolerance compared to corresponding WT mice.

After 14 weeks of HFD feeding, levels of circulating insulin in male adipo‐*Gpr75* ^−/−^ mice were higher (*p* < 0.01) compared to WT (Figure 6F), while insulin levels were significantly lower (*p* < 0.01) in female adipo‐*Gpr75* ^−/−^ compared to WT (Figure 6H). Consequently, HOMA‐IR, an index of insulin resistance, was lower in female adipo‐*Gpr75* ^−/−^ mice as compared to WT (2412 ± 1178 vs. 8932 ± 1640, mean ± SEM, *n* = 9–14, *p* = 0.014), whereas no differences in HOMA‐IR values were observed in male mice (11 070 ± 2217 vs. 16 908 ± 2770 in adipo‐*Gpr75* ^−/−^ and WT mice, respectively; mean ± SEM, *n* = 14–16, *p* = 0.12).

Incretins play a crucial role in maintaining glucose homeostasis. These gut‐derived hormones – namely, glucose‐dependent insulinotropic polypeptide (GIP) and glucagon‐like peptide‐1 (GLP‐1)‐ are released in response to dietary glucose or fat and act to enhance insulin secretion from pancreatic β‐cells [22]. In our study, levels of GIP at the end of the HFD feeding period were not significantly different among male and female genotypes (Figure 6I,L). In contrast, GLP‐1 levels mirrored insulin levels. Hence, GLP‐1 levels following HFD feeding were significantly higher in male adipo‐*Gpr75* ^−/−^ mice compared to WT (Figure 6J), whereas in female adipo‐*Gpr75* ^−/−^ mice, GLP‐1 levels were significantly lower than WT (Figure 6M). Levels of glucagon paralleled GLP‐1 levels, higher in male adipo‐*Gpr75* ^−/−^ compared to WT (*p* < 0.01) and tending lower in female adipo‐*Gpr75* ^−/−^ mice compared to WT (Figure 6K,N).

**FIGURE 7:**
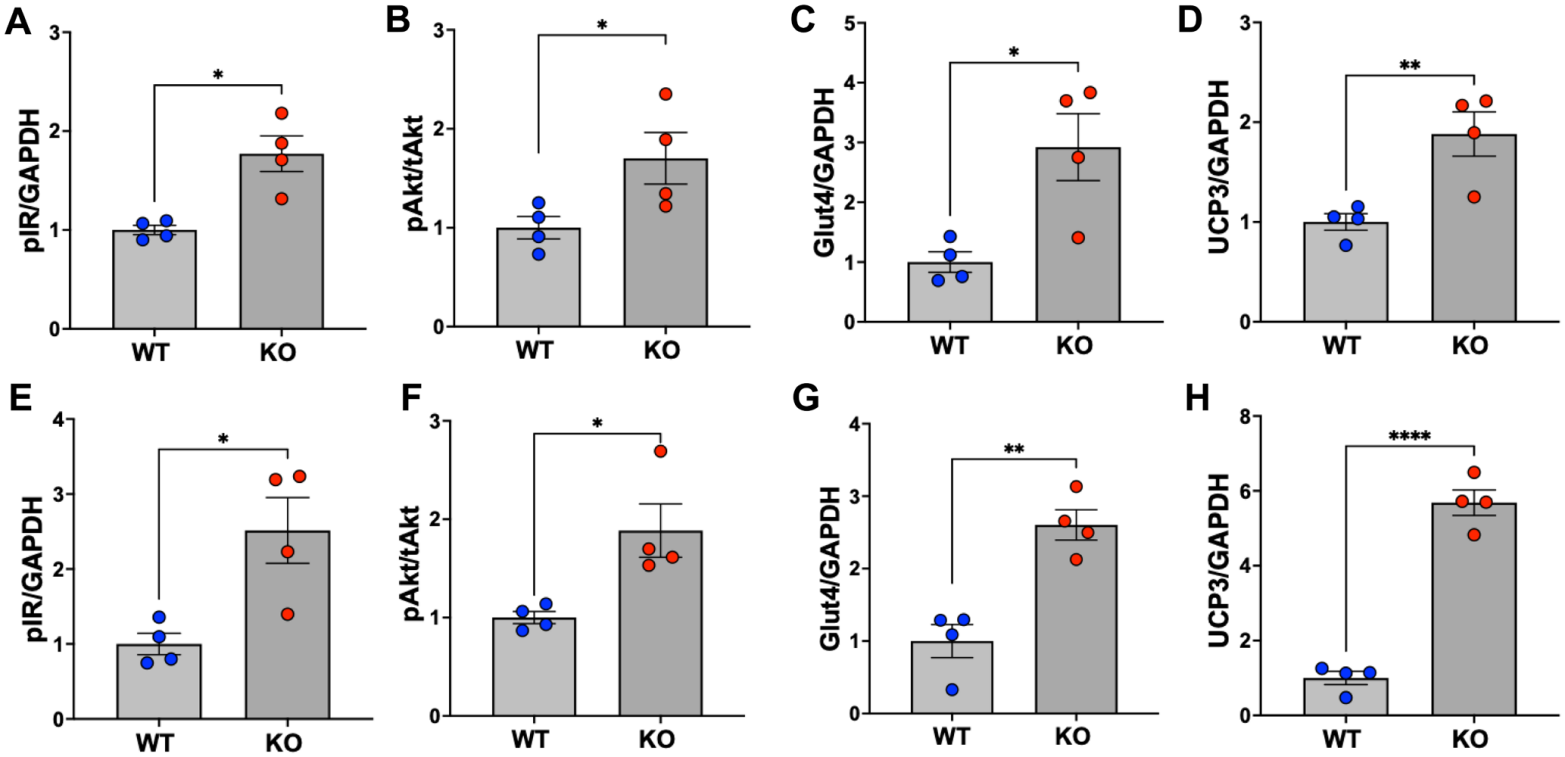
Insulin signaling and thermogenic markers in skeletal muscle from 14‐week‐HFD‐fed male and female WT and adipo‐*Gpr75* ^−/−^ mice. Immunoblots and densitometry analysis relative to GAPDH (37 kDa) of phosphorylated insulin receptor at tyrosine 1334 (pIR, 95 kDa), insulin‐regulated glucose transporter (GLUT4, 55 kDa), mitochondrial uncoupling protein 3 (UCP3, 34 kDa), and phosphorylated AKT at serine 473 (pAKT, 60 kDa) relative to total AKT in male (A–D) and female (E–H) WT and adipo‐*Gpr75* ^−/−^ (KO) mice after 14 weeks of HFD feeding. Results are mean ± SE; *p < 0.05, **p < 0.01 and ***p < 0.001 by unpaired t‐test with Welch's corrections. Images of the immunoblots are included in Data S1.

Maintenance of insulin sensitivity by adipo‐*Gpr75* ^−/−^ mice was also inferred from assessment of key insulin signaling proteins in skeletal muscle. As seen in Figure 7, levels of phosphorylated insulin receptor at tyrosine 1334 and phosphorylated AKT at serine 473 displayed significant increases in male (Figure 7A,B) and female (Figure 7E,F) adipo‐*Gpr75* ^−/−^ mice as compared to WT mice. Similarly, levels of GLUT4, a major insulin‐regulated glucose transporter in muscles were higher in skeletal muscle from male (Figure 7C) and female (Figure 7G) adipo‐*Gpr75* ^−/−^ mice as compared to WT mice. Interestingly, the expression of uncoupling protein 3 (UCP3), a mitochondrial inner membrane protein which regulates oxidative phosphorylation and energy balance in skeletal muscle and is associated with protection against diet‐induced insulin resistance in mice [23], was also higher in male (Figure 7D) and female (Figure 7H) adipo‐*Gpr75* ^−/−^ mice compared to WT mice. Images of the immunoblots are included in the Supplement (Figures S6 and S7).

### 3.5 Effect of Adipocyte *Gpr75* Deficiency on Food Intake, Energy Expenditure, and Activity in Response to HFD

**FIGURE 8:**
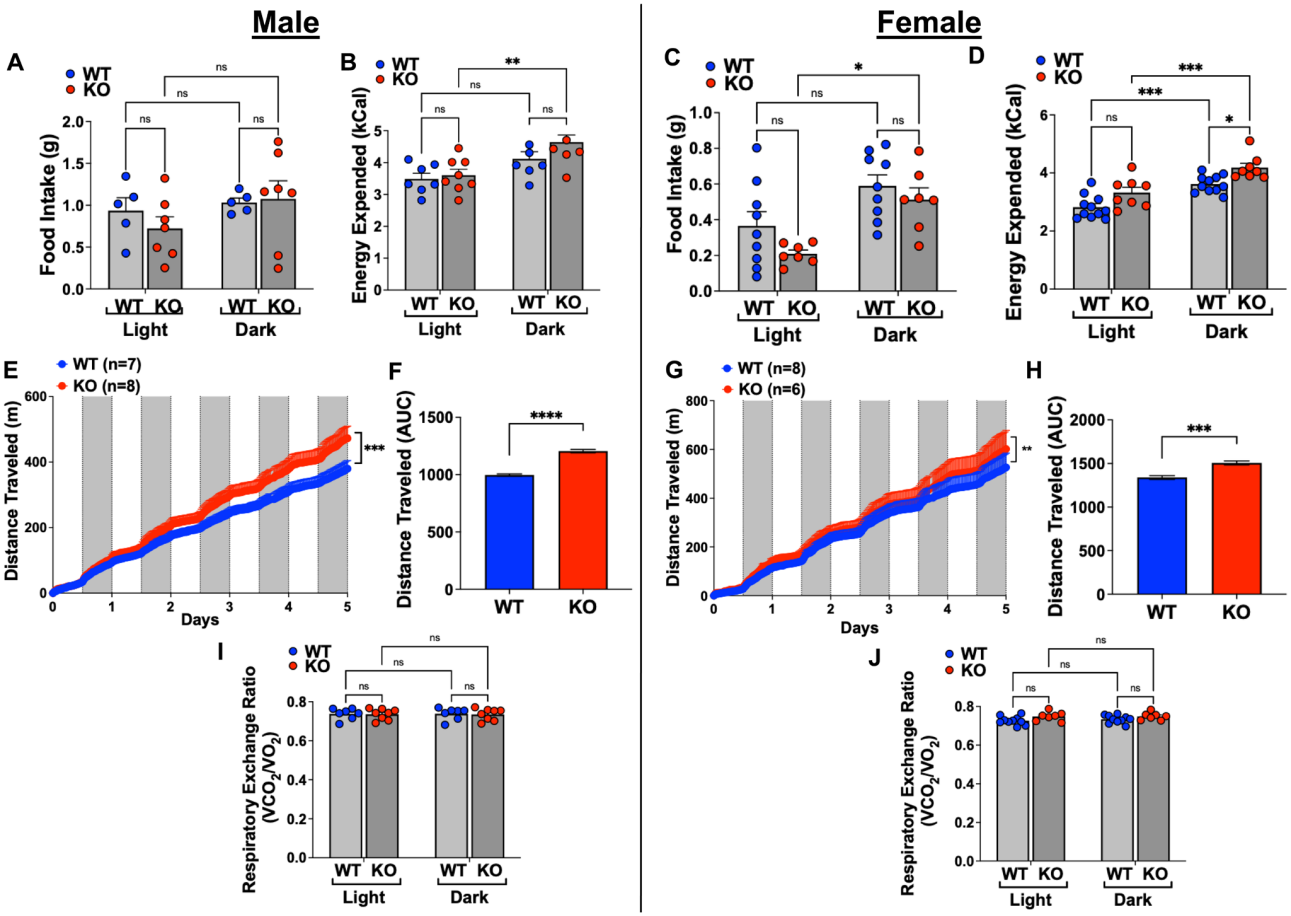
Metabolic parameters in 14‐week‐HFD‐fed male and female WT and adipo‐*Gpr75* ^−/−^ mice. Food intake and energy expenditure in male (A, B) and Female (C, D) mice. Distance travel chart during light (white columns) and dark (gray columns) cycles, and under the curve (AUC) analysis in male (E, F) and Female (G, H) mice. Respiratory Exchange rate (RER) chart and analysis in male (I) and Female (J) mice. All data were collected for 5 days and are presented as average during the light and dark cycles. Results are mean ± SE. Statistical significance between two group was determined by unpaired *t*‐test with Welch's corrections and between multiple groups by two‐way ANOVA with Tukey's multiple comparison (ns, not significant; *p < 0.05, **p < 0.01, ***p < 0.001, and ****p < 0.0001).

Metabolic parameters were assessed before and after HFD feeding using a metabolic cage system and were analyzed separately for light and dark cycles. Baseline metabolic data for male and female WT and adipo‐*Gpr75* ^−/−^ mice on a regular chow diet showed no differences in food intake but significant differences in activity and energy expenditure (Figure S8). Both genotypes had increased food intake and energy expenditure during the dark cycle. However, when measuring physical activity as distance traveled, male and female adipo‐*Gpr75* ^−/−^ mice were more physically active than their WT counterparts. Moreover, respiratory exchange rate (RER) was lower in male adipo‐*Gpr75* ^−/−^ mice (Figure S8) when compared to WT mice in both the light and the dark cycles. In females, RER was not significantly different between the genotypes (Figure S8). RER was higher during the active phase, when food intake and activity are highest, and carbohydrate consumption commonly satisfies energy requirements. Conversely, RER decreased during the inactive phase, when mice are fasting and fatty acids from adipose tissue provide energy. The baseline metabolic data showing that adipo‐*Gpr75* ^−/−^ mice are more active than corresponding WT suggest a direct effect of GPR75 on metabolism; however, a thorough examination of central vs. peripheral contributions of GPR75 needs to be further explored and would require the development of new animal models (inducible on/off). Following 14 weeks of HFD feeding, metabolic analysis showed no significant differences in food intake across the experimental groups (Figure 8A,C). Energy expenditure (kCal) was higher in male adipo‐*Gpr75* ^−/−^ mice during the dark cycle, but no differences between WT and adipo‐*Gpr75* ^−/−^ mice were observed (Figure 8B). Female aKO mice showed the same trend of increase in energy expenditure during the dark cycle, which was significantly different from corresponding WT mice (Figure 8D). Importantly, both male (Figure 8E,F) and female (Figure 8G,H) adipo‐*Gpr75* ^−/−^ mice were significantly more active than their corresponding WT mice. Respiratory exchange rate was expectedly similar between the genotypes as the main energy source during the experiment was diet enriched in fat (Figure 8I,J). Analysis of energy expenditure by ANCOVA using body mass or lean mass as covariates indicated no significant interactions (Figure S9A,B), suggesting that the observed differences in energy expenditure between the groups are the consequence of the differences in their body composition.

## 4 Discussion

Adipocytes play an active role in regulating metabolism. However, in obesity, their function becomes maladaptive, leading to excessive fat accumulation, systemic inflammation, insulin resistance, and broader metabolic disturbances. This dysfunction is a key driver of obesity‐related conditions such as type 2 diabetes, cardiovascular disease, and non‐alcoholic fatty liver disease. GPR75, recently identified as an obesity‐associated gene [3], is expressed in adipocytes, and its expression appears to increase in response to an HFD [7]. Previous reports indicate a clear link between global deletion of *Gpr75* and protection against diet‐induced obesity and metabolic syndrome [3, 7, 8, 9]. The mechanisms underlying this protection are yet to be fully explored. Several reports suggested that given the high expression of GPR75 in the brain, central mechanisms that target appetite and energy balance are involved. Powell et al. [6] reported that both male and female *Gpr75* ^*−/−*^ mice are thin at weaning and hypophagic on a chow or HFD diet as the thin phenotype becomes more exaggerated, suggesting that GPR75 influences body composition by regulating energy intake. A study by Jiang et al. [9], using a pair feeding experiment, suggested that decreased food intake was the main contributor to the lean phenotype of the *Gpr75* ^*−/−*^ mice. A recent study reported that mice with brain‐specific Gpr75 deletion, using the Nestin‐Cre, were resistant to diet‐induced obesity, primarily through suppressed food intake and modest increases in energy expenditure [24]. Of note, Hossain et al. [7] reported that there were no differences in food intake between WT and Gpr75^−/−^ mice when fed either chow diet or HFD, whereas Leeson‐Payne et al. [8] found no genotypic differences in food intake on a chow diet and that *Gpr75* ^*−/−*^ mice adjusted caloric intake to retain energy balance when fed HFD. Hence, the role of GPR75 in the regulation of energy balance is still not fully understood and may be heavily dependent on the genetic underpinnings of each animal model.

Nevertheless, GPR75 expression is not limited to the brain [25]. While the brain plays a well‐established role in regulating energy balance, it is important to highlight the critical contribution of adipose tissue. As a source of adipokines that influence appetite regulation in the brain, adipose tissue actively participates in maintaining energy homeostasis. This suggests that peripheral mechanisms—particularly within adipose tissue—may contribute to the protective effects observed with global GPR75 deletion against diet‐induced obesity. In view of the central role of adipocytes in the pathogenesis of obesity, we selected to generate mice that have adipocyte‐specific *Gpr75* genetic disruption to determine whether these mice are protected from diet‐induced obesity.

The key finding of this study is that deleting *Gpr75* specifically in adipocytes offers protection against diet‐induced obesity across several fronts. Moreover, our data show that this protection is not due to reduced food intake but instead is linked to sustained physical activity, which well correlated with reduced adiposity, improved glucose metabolism, and lower levels of inflammation. Notably, this protection, unlike that reported for the global knockout [7] or the hypothalamic neuronal cilia specific deletion [9], demonstrated a striking sexual dimorphism which is summarized in Table S1. Specifically, female adipo‐*Gpr75* ^−/−^ mice showed significantly attenuated weight gain compared to WT mice after HFD‐feeding, while male adipo‐*Gpr75* ^−/−^ mice gained weight similar to that of WT mice. However, in both male and female adipo‐*Gpr75* ^−/−^ mice, HFD‐driven impairments in glucose handling and insulin sensitivity were largely attenuated compared to WT as evidenced by lower fasting blood glucose and rapid glucose clearance from the blood with exogenous glucose administration as well as preserved skeletal muscle insulin signaling. Yet, maintenance of glucose homeostasis in adipo‐*Gpr75* ^−/−^ mice in response to HFD appeared to be different between the sexes. In comparison to WT mice, male adipo‐*Gpr75* ^−/−^ mice displayed hyperinsulinemia with higher levels of GLP‐1, whereas female adipo‐*Gpr75* ^−/−^ mice had lower insulin and GLP‐1 levels. This divergence may be driven by sex‐specific factors, functioning as a compensatory mechanism to maintain glucose homeostasis in males and as a protective strategy to limit excessive insulin secretion in females.

The divergence in GLP‐1 and insulin response between male and female adipo‐*Gpr75* ^−/−^ is intriguing. The improved glucose handling seen in the adipo‐*Gpr75* ^−/−^ mice can result from increased insulin secretion or increased insulin sensitivity or both. Therefore, the increased insulin and GLP‐1 in males likely reflect an insulin‐secretory compensation, while the lower insulin and GLP‐1 in females paired with better glucose handling is consistent with enhanced peripheral insulin sensitivity, suggesting that less insulin is required to clear glucose. An inducible, tissue‐specific model would assist in the elucidation of these relationships. As for potential interactions between GLP‐1 and GPR75 at the levels of β‐cells, there are several reports that allude to such interactions. Liu et al. [26] demonstrated islet expression of GPR75 and suggested that it is coupled to stimulation of insulin secretion and improved glucose homeostasis. Additionally, a recent study reported that the GLP‐1 agonist liraglutide increases the expression of GPR75 in murine NIT‐1 pancreatic beta cells [27]. However, these studies did not provide evidence for direct interactions between GLP‐1 and GPR75. In the absence of direct evidence linking GLP‐1 and adipocyte GPR75, the changes in incretins may be secondary to systemic metabolic alterations rather than adipocyte‐gut axis.

Adipose tissue dysfunction—characterized by immunological changes (i.e., changes in activation state of various leukocyte populations, and therefore, altered levels of cytokines and chemokines)—is widely accepted as a major factor in the pathogenesis of obesity‐related diseases such as type 2 diabetes [28]. Pro‐inflammatory cytokines, particularly TNFα, directly inhibit normal insulin signaling through activation of JNK or IKKβ/NF‐κB signaling pathways [29]. Our data indicates that both male and female adipo‐*Gpr75* ^−/−^ mice were largely protected against adipose inflammation as evidenced by lower circulating levels of the pro‐inflammatory adipokines, leptin and resistin [30] and the lower expression of the inflammatory markers TNFα and NF‐kB within the adipose tissue. Likewise, adipocyte hypertrophy as a result of fat accumulation is a key process in response to HFD which leads to adipocyte dysfunction and eventually to insulin resistance [31]. In our study, adipocytes of both male and female adipo‐*Gpr75* ^−/−^ mice were protected from the detrimental effects of HFD as demonstrated by attenuated adipocyte hypertrophy. In adipo‐*Gpr75* ^−/−^ males, which gained fat mass similar to WT, this protection may also be derived from the increased TREM‐2 positive LAM which are known to accumulate within adipose tissues in response to HFD. These specialized macrophages are attracted to the adipose tissue primarily by extracellular lipids released from dying or stressed adipocytes and contribute to metabolic health by promoting anti‐inflammatory lipid clearance. The relationship between inflammatory cytokines such as TNFα and the TREM2‐LAM is seemingly paradoxical and is not straightforward. However, several reports have demonstrated in models of metabolic and cardiovascular diseases that TREM2 inhibits the activation of NF‐κB and the production of inflammatory cytokines including TNFα [32, 33]. Others used TREM2 siRNA, TREM2 overexpression, and TREM2 knockout mice to show that TREM2 functions to inhibit cytokine production by macrophages in vitro and in vivo [34, 35, 36]. It is possible that the reduction of inflammatory markers within the adipose tissue is a consequence of accumulated TREM2‐LAM, which are attracted to the adipose tissue by an excess of extracellular lipids.

The observation that *Gpr75* deficiency provides greater benefits to females than males has been previously reported in the global *Gpr75* knockout model [7]. In fact, the female adipo‐*Gpr75* ^−/−^ phenotype closely resembles that of the global *Gpr75* knockout and quantitatively corresponds to the female heterozygous *Gpr75* ^+/−^ mice [3, 7]. In the present study, female adipo‐*Gpr75* ^−/−^ mice were lean and demonstrated a phenotype characterized by decreased insulin, GLP‐1, and glucagon levels after HFD feeding. Female adipo‐*Gpr75* ^−/−^ mice maintained insulin sensitivity and appeared to be leptin sensitive during the HFD feeding period. They were not hypo or hyperphagic, and their adipocytes seemed to be metabolically healthy as evidenced by the adiponectin to leptin ratio. The female phenotype may be explained by the action of estrogen, which is known to inhibit lipogenesis, stimulate catecholamine‐induced lipolysis, and possess anti‐inflammatory effects in many metabolic diseases [37, 38, 39]. Estrogen is also known to affect metabolic homeostasis in BAT by increasing thermogenic gene expression leading to increased energy expenditure [40]. Indeed, female adipo‐*Gpr75* ^−/−^ mice exhibited smaller VAT and SAT adipocytes that expressed higher levels of UCP1 when compared to WT adipocytes, suggesting better lipid handling and less hypertrophy. Moreover, the number of BAT adipocytes in response to HFD appeared to greatly increase in female adipo‐*Gpr75* ^−/−^ mice, suggesting increased thermogenic capacity. Unquestionably, the role of estrogen in this model needs to be extensively investigated using tools such as ovariectomy and hormone replacement.

On the other hand, deletion of Gpr75 in adipocytes did not prevent weight gain in male mice in response to HFD. However, similar to the female KO mice, HFD‐fed male adipo‐*Gpr75* ^−/−^ mice showed reduced adiposity and adipocyte hypertrophy and exhibited a metabolically healthy obese phenotype with lower levels of inflammation compared to their WT counterparts [41, 42]. This may be the result of a GLP‐1‐driven metabolic cascade that shields from obesity‐induced hyperglycemia and insulin resistance. The attenuation of inflammation together with increased capacity to handle lipid overload—via the accumulation of TREM2 positive LAM within adipose tissues and increased UCP1 expression—and the concerted decrease in leptin and increase in the GLP‐1‐insulin circuit may constitute this unique protection in the male adipo‐*Gpr75* ^−/−^ mice. Additionally, there are studies suggesting a role for androgen in enhancing muscle glucose uptake and amplifying GLP‐1 secretion [43] which is seen in the male KO mice. Whether androgen contributes to this adaptive mechanism needs to be further investigated using tools such as castration and hormone replacement.

The mechanisms by which adipocyte‐derived GPR75 contribute to DIO‐driven obesity and metabolic diseases are yet to be fully explored. Studies with the reported GPR75 ligand, 20‐hydroxy‐eicosatetraenoic acid (20‐HETE), may provide some clues. 20‐HETE is a vasoactive and pro‐inflammatory lipid mediator. The actions of 20‐HETE among others include stimulation of smooth muscle contraction, eNOS uncoupling and endothelial dysfunction, activation of the NF‐κB‐inflammatory program and increased production of inflammatory cytokines, activation of the renin‐angiotensin system, and inhibition of mitochondrial biogenesis [44]. These actions of 20‐HETE and their contribution to cardiovascular, cerebrovascular, and metabolic disease have been associated with the high‐affinity binding to and activation of GPR75 by 20‐HETE [25, 44, 45, 46, 47]. Regarding obesity, plasma and tissue levels of 20‐HETE increased in animal models of obesity and metabolic syndrome and strongly correlated with BMI in humans [48, 49, 50, 51, 52]. 20‐HETE stimulates lipid accumulation during adipogenesis in vitro [53], and a role for 20‐HETE‐GPR75 pairing in metabolic dysfunction has been suggested [14]. 20‐HETE production in white adipose tissue is 2–4‐fold higher in HFD‐fed mice than in mice fed a regular chow [54, 55]. We and others have shown that 20‐HETE impairs insulin receptor signaling [54, 55, 56, 57], and that overexpression of 20‐HETE synthases in mice is associated with exacerbated diet‐driven weight gain, hyperglycemia, and insulin resistance [54, 55, 58]. Furthermore, using GPR75 receptor blockers, a causal relationship between 20‐HETE and insulin resistance in HFD‐driven obesity, and between 20‐HETE levels and impaired insulin signaling in adipose tissue, skeletal muscles, and liver, has been demonstrated [54, 55]. Based on its pro‐inflammatory properties (NF‐κB activation, induction of pro‐inflammatory cytokines and stimulation of oxidative stress) along with its ability to interfere with insulin signaling and mitochondrial biogenesis, adipocyte‐derived 20‐HETE‐GPR75 pairing probably contributes to obesity‐driven insulin resistance and metabolic dysfunction.

The chemokine RANTES/CCL5, which was also proposed a ligand/interactor for GPR75 [59, 60], has been shown to play a role in the development of obesity primarily through its pro‐inflammatory actions [61, 62]. Several studies showed that CCL5 and its primary receptor CCR5 are upregulated in white adipose tissue driving obesity‐induced inflammation and insulin resistance by recruiting macrophages and CD8+ T‐cells. Moreover, deletion of *Ccr5* or *Ccl5* in mice protects against diet‐induced insulin resistance [61, 62]. In contrast, a recent study suggested that CCL5 deficiency in mice exacerbates adipose tissue inflammation and impaired insulin sensitivity [63]. The role of CCL5‐GPR75 pairing in obesity is unknown. A study by Liu et al. suggested that CCL5‐GPR75 pairing is involved in β‐cell insulin secretion in vitro [26]; the in vivo implication of such action/pairing is yet to be explored. In our study, we did not find differences in levels of CCL5 following HFD feeding between the genotypes suggesting that the observed phenotypes are unlikely to be affected by alterations in CCL5 signaling.

In summary, this study demonstrates that adipocyte‐specific loss of GPR75 expression is sufficient to protect against metabolic disorders linked to diet‐induced obesity. However, the observed phenotypes likely reflect integrated effects across peripheral and central pathways. Hence, cell‐specific deletion of *GPR75* in the central nervous system or vascular compartments may be required to recapitulate and unmask the cell‐ or tissue‐specific contributions necessary for complete protection from diet‐induced obesity. Nevertheless, GPR75 appears to be a crucial regulator of adipocyte health, and its inhibition could offer a promising therapeutic approach for obesity and metabolic syndrome.

## Author Contributions

S.H., V.G., and M.L.S. conceptualized and initiated the project and were involved in planning and designing experiments, analyzing data, writing the first draft, reviewing, and editing up to the final draft. S.H., E.V., D.D., and C.L. performed the experiments, assisted in analyzing, and interpreting data and in generating figures. D.K., N.S., A.C., and C.W. performed various assays and assisted with carrying out the experiments. A.G. and D.C.Z. developed, generated, and validated the transgenic mice used in this study. M.L.S. supervised the implementation of all experiments and the completion of the study.

## Funding

This work was supported by grants from the National Institutes of Health (HL139793 and TR004478).

## Disclosure

The authors declare that no Generative AI was used in the creation of this manuscript.

## Conflicts of Interest

The authors declare no conflicts of interest.

## Supporting information


**Data S1:** Supporting information.

## Data Availability

The data that support the findings of this study are available in the Materials and Methods, Results, and Data S1 of this article.
