# Supplementary material for: Gpr75 Deletion in Adipocytes Protects From Diet‐Induced Obesity: Changes in Glucose Homeostasis and Inflammatory Responses
Source: FASEB J. 2026 Feb 18;40(4):e71579. doi: 10.1096/fj.202504597R (PMC12916076; doi:10.1096/fj.202504597R)
Supplement: Supplementary file 1 — Data S1: Supporting information. [file FSB2-40-e71579-s001.pdf]

Supplementary Material

Hossain et al., *Gpr75* Deletion in Adipocytes Protects from Diet Induced Obesity: Changes in Glucose Homeostasis and Inflammatory Responses

Category	Parameter	Male WT	Male KO	Female WT	Female KO	Trend vs. WT	
Metabolic Phenotypes	Δ Body Weight (g)	17.94±1.00 (17)	19.52±1.56 (14)	16.09±1.33 (18)	8.22±1.11 (11) c	♂ ↔	♀↓
	Fasting Blood Sugar (mg/dL)	216.43±14.71 (7)	133.00±11.33 (8) d	153.13±14.28 (8)	110.82±4.38 (11) b	♂ ↓	♀↓
	W14 GTT AUC (mg/dL x min	2,404.00±140.00 (13)	1,626.00±254.00 (14) a	2,553.00±105.90 (3)	1,701.00±114.00 (4) b	♂ ↓	♀↓
	SAT Adipocyte Size (um^2)	8,181.46±719.10 (5)	7,665.39±1,079.08 (5) a	7,969.93±493.87 (6)	3,584.05±480.06 (6) a	♂ ↓	♀↓
	VAT Adiopcyte Size (um^2)	8,020.75±543.62 (6)	6,051.76±792.33 (8) b	8,188.23±350.78 (6)	3,867.23±426.59 (6) b	♂ ↓	♀↓
Plasma Cytokines & Inflammatory Markers	GLP-1 (pg/ml)	31.85±6.10 (7)	74.19±10.41 (10) b	101.16±19.81 (16)	28.67±4.90 (15) b	♂ ↑	♀↓
	GIP (pg/ml)	508.13±45.83 (16)	608.25±38.84 (14)	456.12±34.02 (14)	336.51±69.27 (12)	♂ ↔	♀ ↔
	Insulin (pg/ml)	1,236.54±172.10 (16)	3,335.93±441.50 (17) c	1,143.07±159.24 (16)	569.26±97.60 (15) b	♂ ↑	♀↓
	Adiponectin (ug/ml)	3.97E+07±8566691.478 (7)	2.25E+07±4931585.222 (9) a	2.88E+07±1836232.535 (9)	2.06E+07±7262144.897 (6)	♂ ↓	♀ ↔
	Leptin (pg/ml)	13,580.31±1,790.38 (11)	9,534.33±981.22 (17) a	7,463.17±715.81 (16)	3,892.05±736.14 (15) b	♂ ↓	♀↓
	Ghrelin (pg/ml)	1,927.56±381.82 (7)	862.30±179.61 (10) a	1,463.97±188.93 (18)	1,314.23±337.41 (15)	♂ ↓	♀ ↔
	Glucagon (pg/ml)	157.87±23.27 (17)	291.70±37.21 (18) b	159.39±26.56 (18)	114.88±22.45 (15)	♂ ↑	♀ ↔
	Resistin (pg/ml)	39,535.73±5,240.35 (7)	24,250.48±1,687.15 (10) b	34,895.24±3,661.10 (16)	21,722.85±3,790.03 (15) a	♂ ↔	♀↓
	CCL5/RANTES (pg/ml)	50.36±9.29 (7)	56.84±7.26 (10)	165.62±73.22 (8)	34.42±6.69 (7)	♂ ↔	♀↓
	TNF-α (pg/ml)	11.51±2.14 (7)	14.74±2.92 (10)	5.48±0.75 (9)	0.58±0.38 (7)	♂ ↔	♀ ↔
Metabolic Cage Parameters	Total Distance Traveled (m)	197.9±6.97 (7)	239±8.75 (8) c	265.5±10.06 (8)	298.8±11.13 (6) b	♂ ↑	♀↑
	Energy Expenditure (kCal)						
	Light	3.49±0.18 (7)	3.61±0.19 (8)	2.82±0.12 (11)	3.33±0.18 (8)	♂ ↔	♀ ↔
	Dark	4.12±0.22 (7)	4.64±0.23 (8)	3.62±0.09 (11)	4.19±0.15 (8) a	♂ ↔	♀↑
	Food Intake (g)						
	Light	0.93±0.16 (5)	0.72±0.14 (7)	0.37±0.08 (9)	0.21±0.02 (7)	♂ ↔	♀ ↔
	Dark	1.03±0.05 (5)	1.08±0.22 (7)	0.59±0.06 (9)	0.51±0.07 (7)	♂ ↔	♀ ↔

Supplementary Table 1: Summary of major findings and differences observed between male and female mice in their response to HFD feeding.

All values are shown as Mean±SEM (n); a, $p<0.05$; b, $p<0.01$; c, $p<0.001$; d, $p<0.0001$
Arrows (↑, ↓, ↔) indicate the direction of change in KO mice compared with WT and mirror the statistical results (p-values) reported in the manuscript.

↔ indicates no significant difference between KO and WT groups

↓ indicates reduced levels in KO mice compared with WT

↑ indicates increased levels in KO mice compared with WT

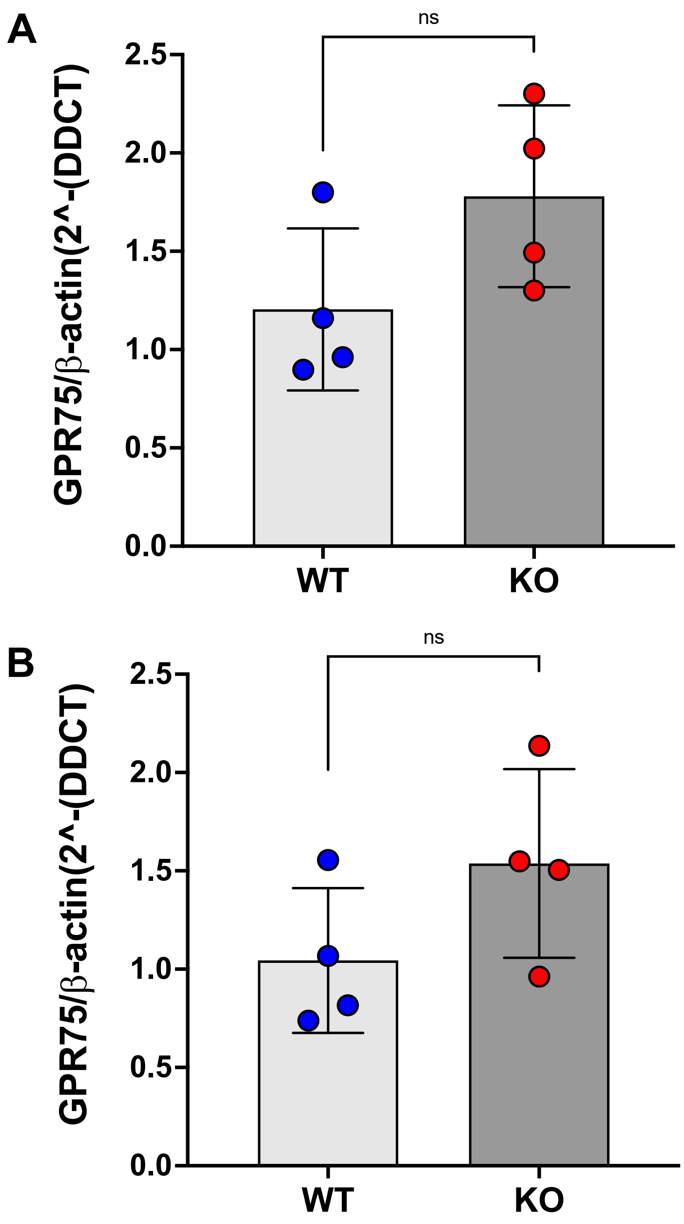

Supplementary Figure 1: Gpr75 mRNA levels in brain (A) and liver (B) of male WT and KO mice. Statistical significance between the two group was determined by unpaired t test with Welch's corrections (ns, not significant).

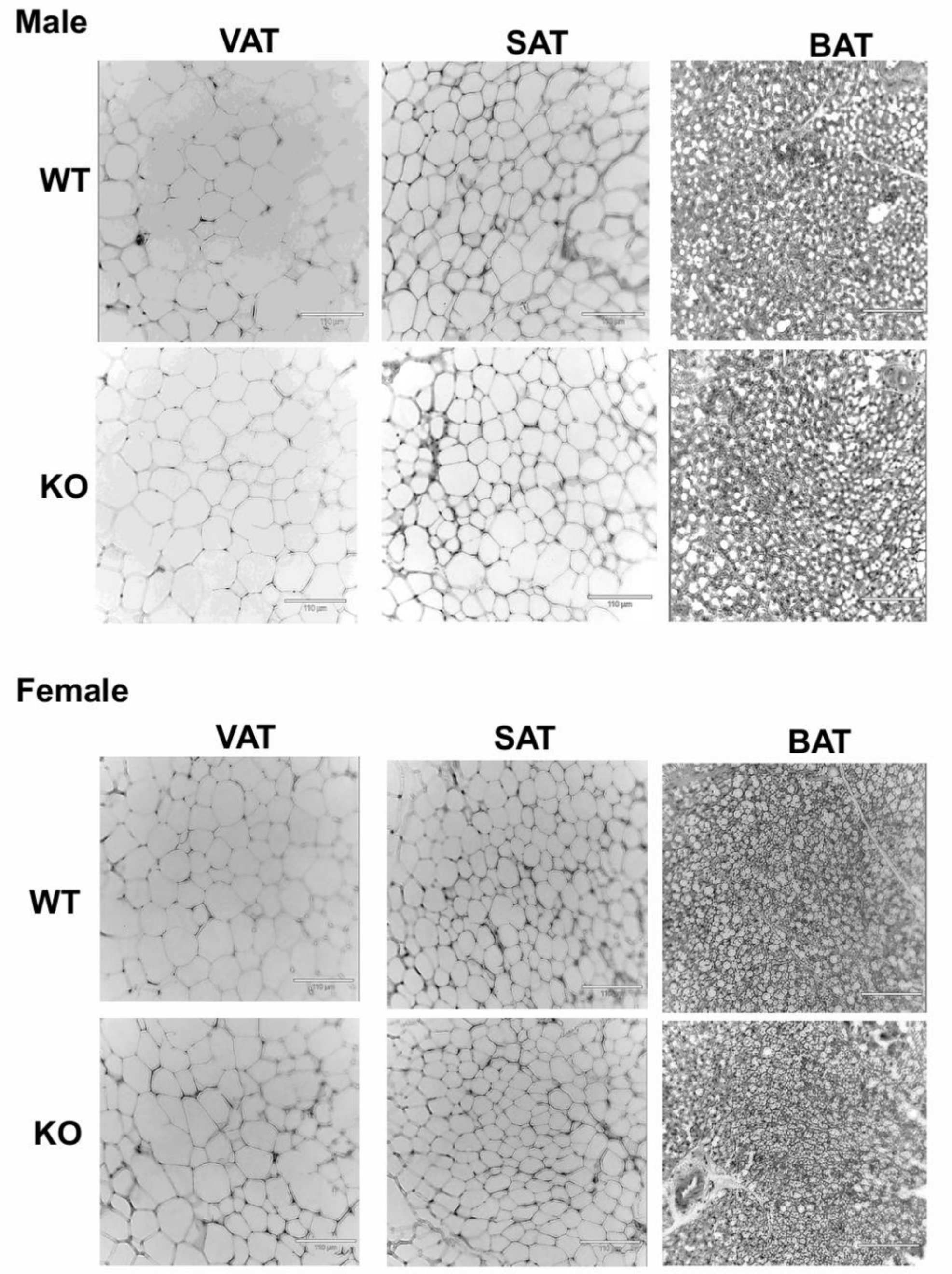

Supplementary Figure 2: Representative images of H&E staining in visceral (VAT), subcutaneous (SAT) and brown (BAT) adipose tissues of male and female WT and KO mice at the onset of HFD feeding. All images were taken at 20X magnification with scale bar represent 110 µm. There was no difference in adipocyte size between the genotypes at baseline. In males, size of VAT and SAT adipocytes averages 2447±200 and 1256±114 μm^2, respectively, and in females 1889±98 and 1202±87 μm^2, respectively.

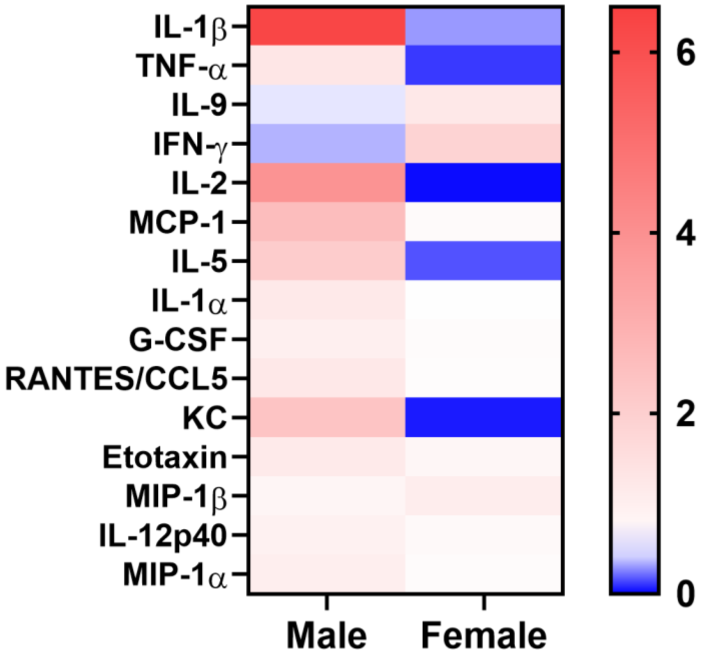

<u>Supplementary Figure 3</u>: Heat map depicting levels of plasma cytokines in HFD-fed male and female KO mice as fold change from corresponding WT mice.

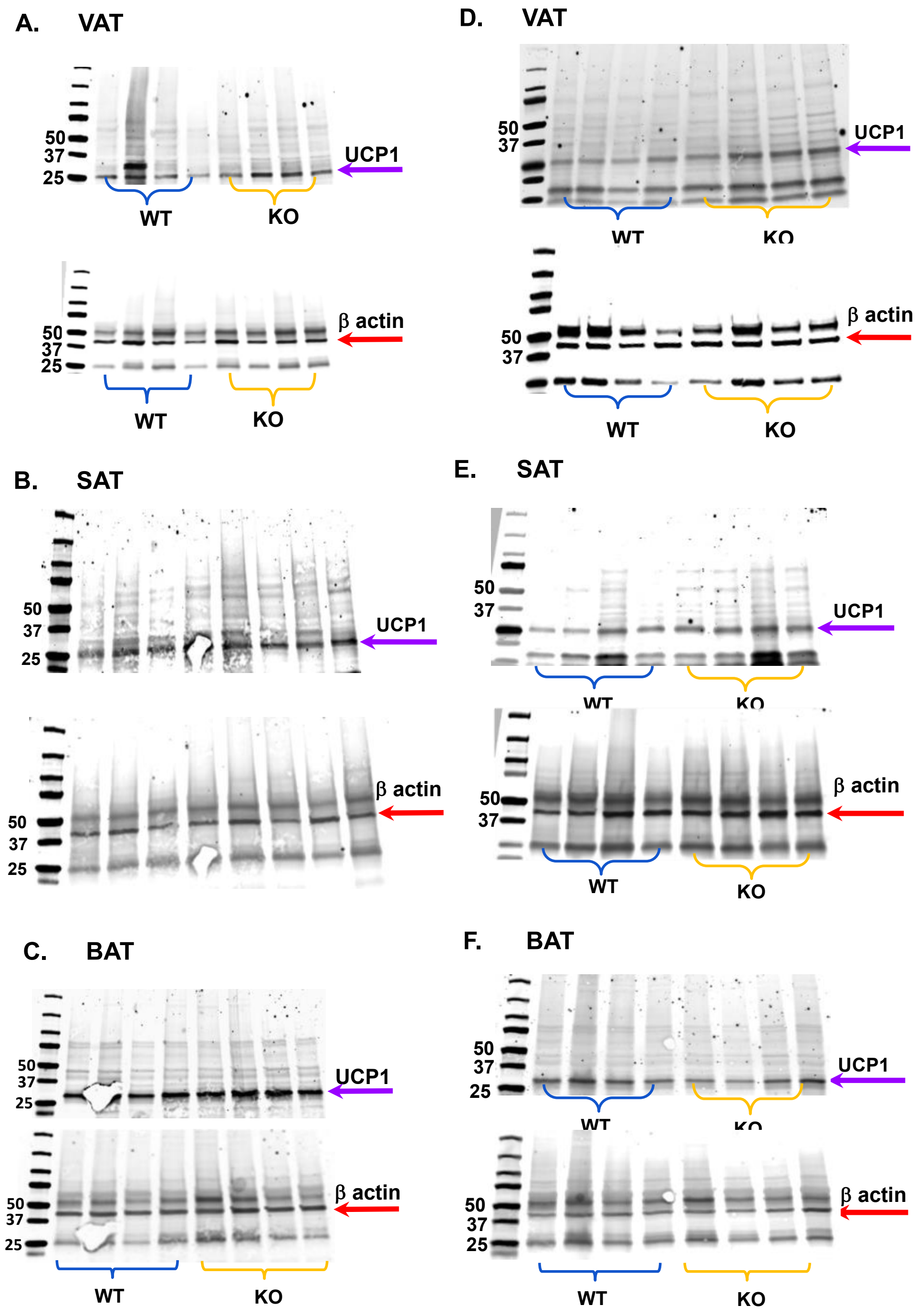

Supplementary Figure 4: Representative western blot images of UCP1 in VAT, SAT and BAT of male (A, B and C, respectively) snf female (D, E and F, respectively) WT and KO mice. The molecular weights of UCP1 and β-actin are 30 kDa and 42 kDa, respectively

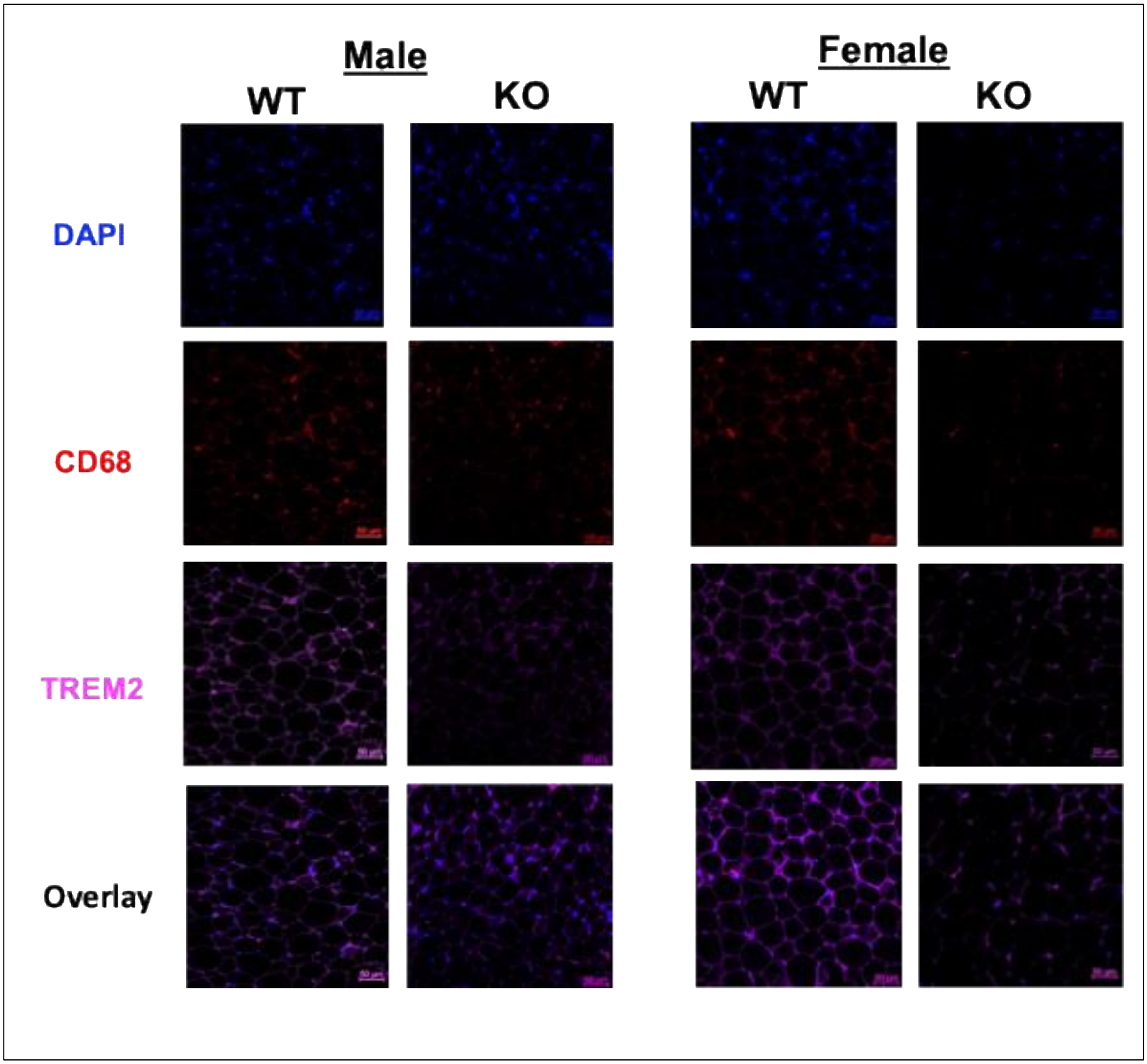

Supplementary Figure 5: Representative immunofluorescence images of visceral adipose tissue (VAT) from male and female WT and KO mice at baseline before HFD feeding. Images were taken at 20X, size bar is 50 µm.

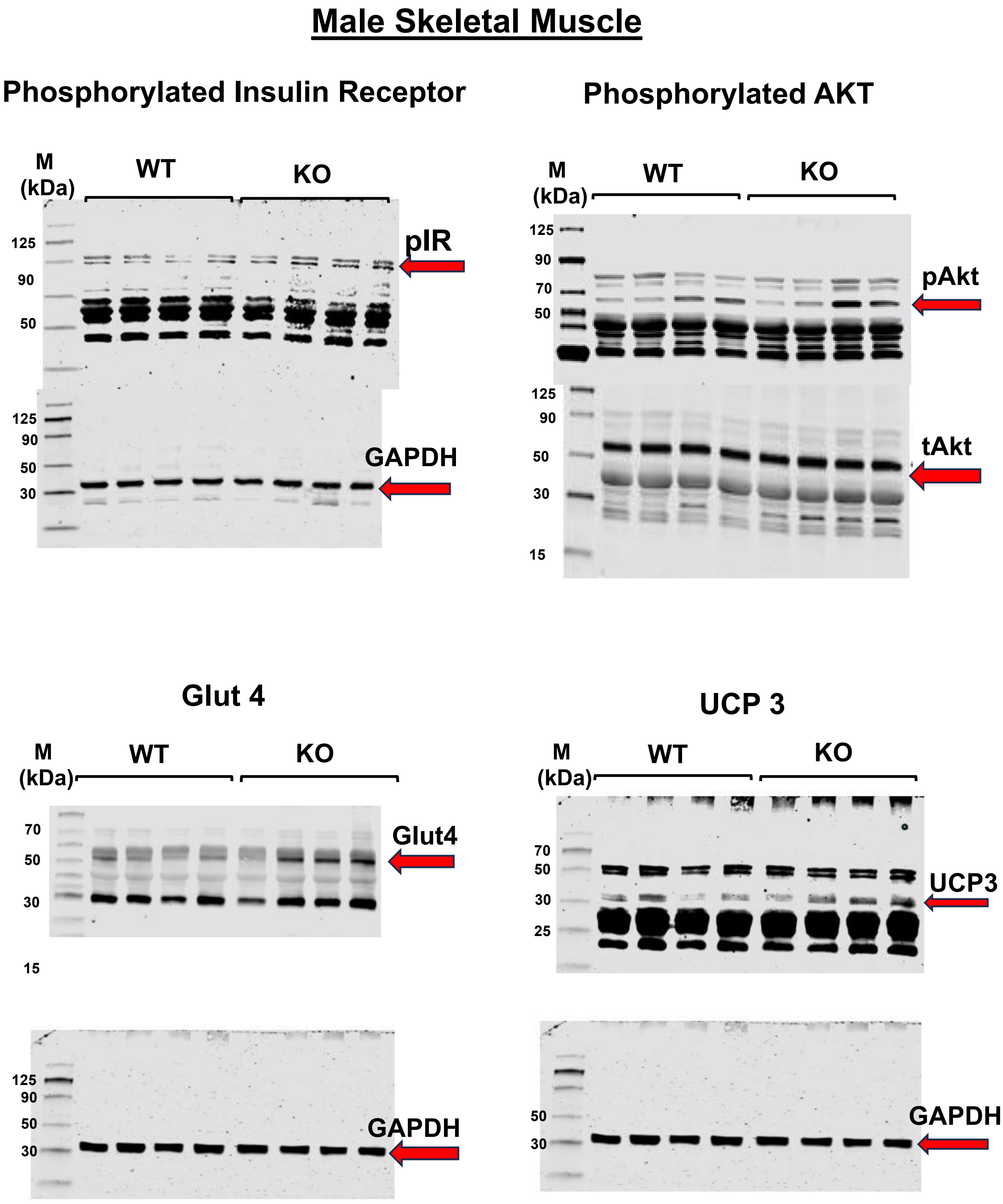

Supplementary Figure 6: Immunoblots of skeletal muscle from HFD-fed male WT and KO mice with antibodies against phosphorylated insulin receptor (pIR), AKT and phosphorylated AKT (pAKT), Glut 4 and UCP3. The Glut 4 and UCP3 immunoblots were done on the same membrane and therefore GAPDH is the same.

Female Skeletal Muscle

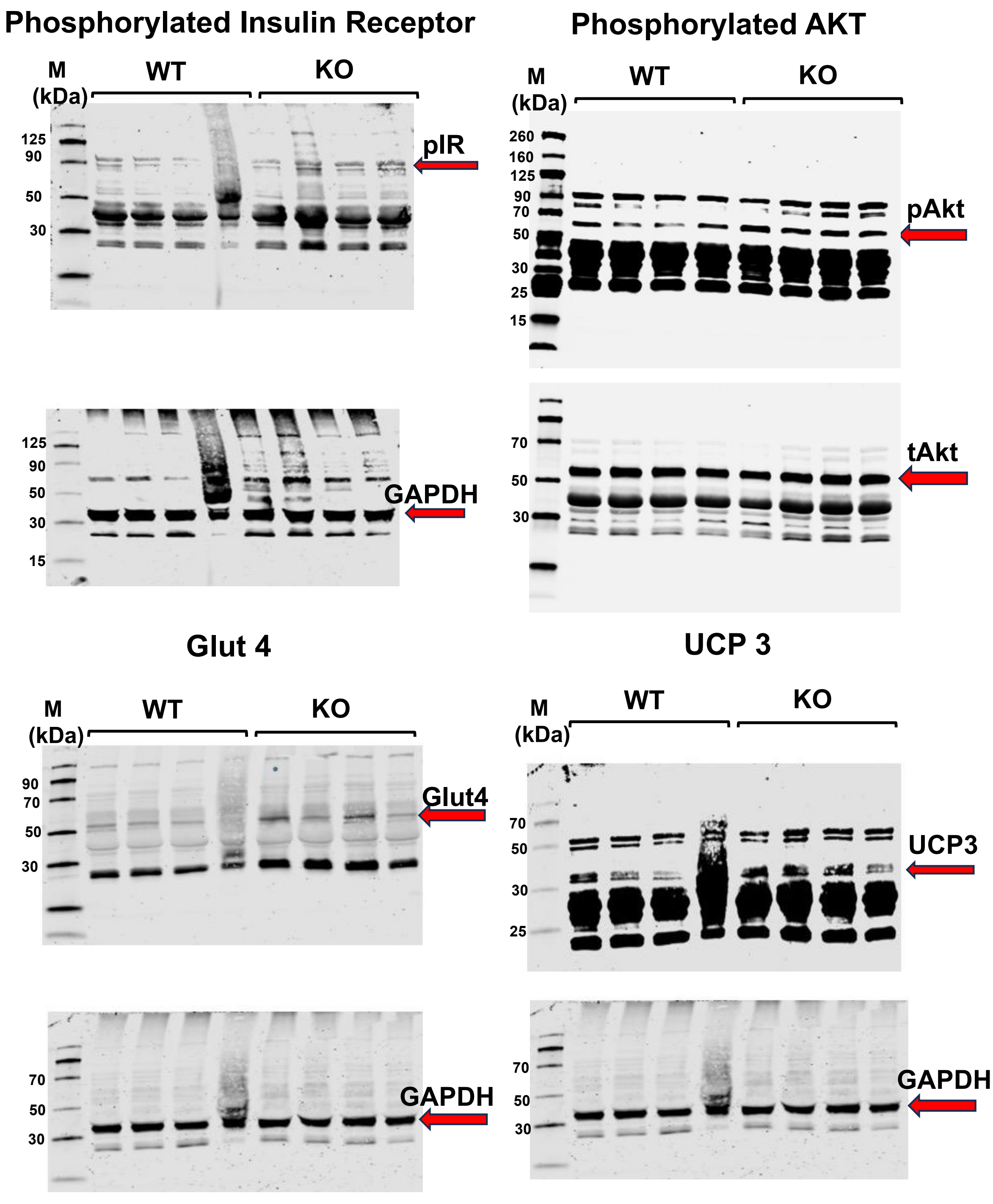

Supplementary Figure 7: Immunoblots of skeletal muscle from HFD-fed female WT and KO mice with antibodies against phosphorylated insulin receptor (pIR), AKT and phosphorylated AKT (pAKT), Glut 4 and UCP3. The Glut 4 and UCP3 immunoblots were done on the same membrane and therefore GAPDH is the same.

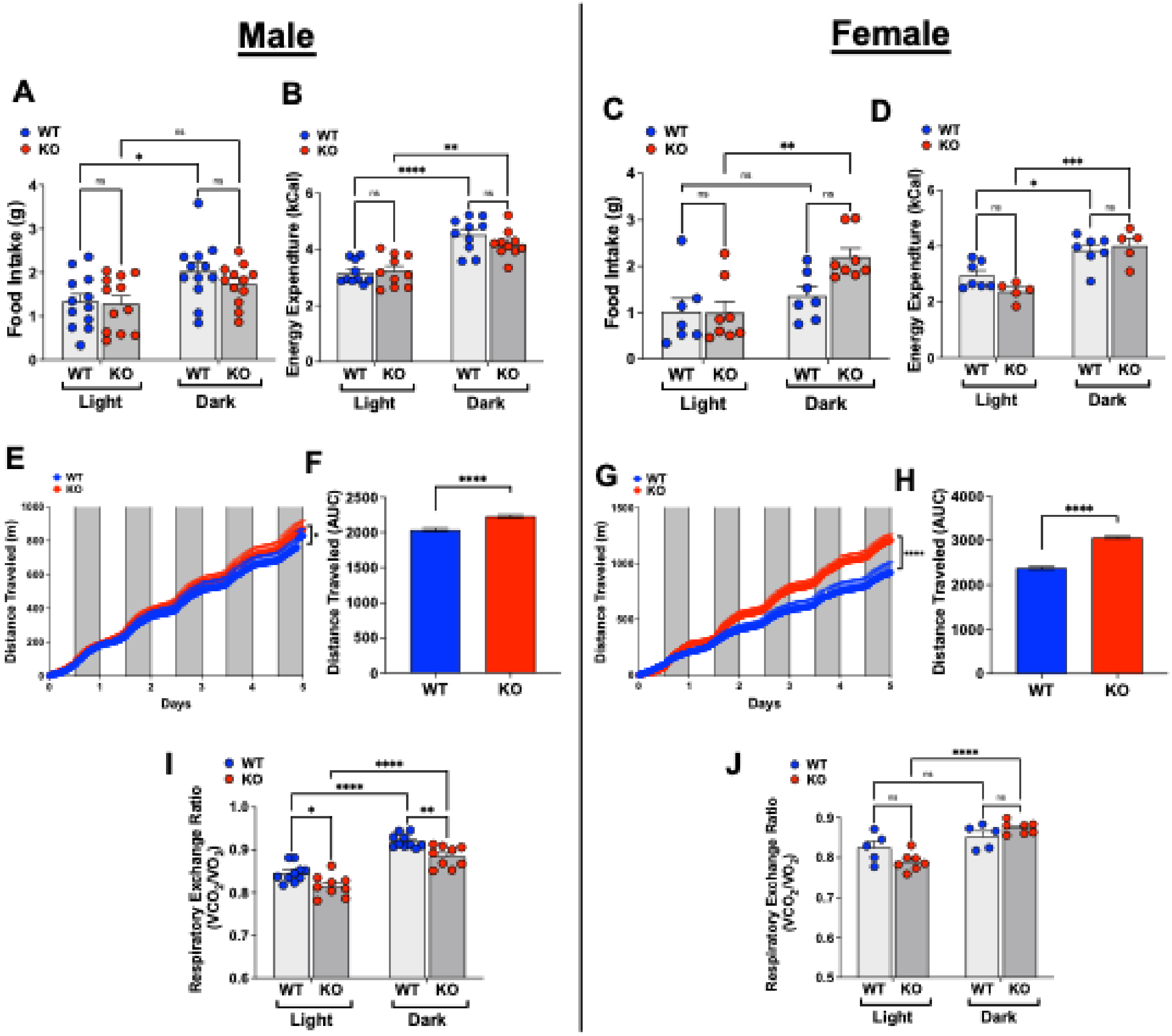

Supplementary Figure 8: Baseline metabolic parameters in male and female WT and KO mice. Food intake and energy expenditure in male (**A,B**) and Female (**C,D**) mice. Distance travel chart and analysis in male (**E,F**) and Female (**G,H**) mice. Respiratory Exchange rate (RER) analysis in male (**I**) and Female (**J**) mice. All data were collected for 5 days and are presented as daily average during the light and dark cycles. Results are mean±SE; ns, not significant; *p<0.05, **p<0.01, ***p<0.001 and ****p<0.0001 by two-way ANOVA with Tukey's multiple comparison test.

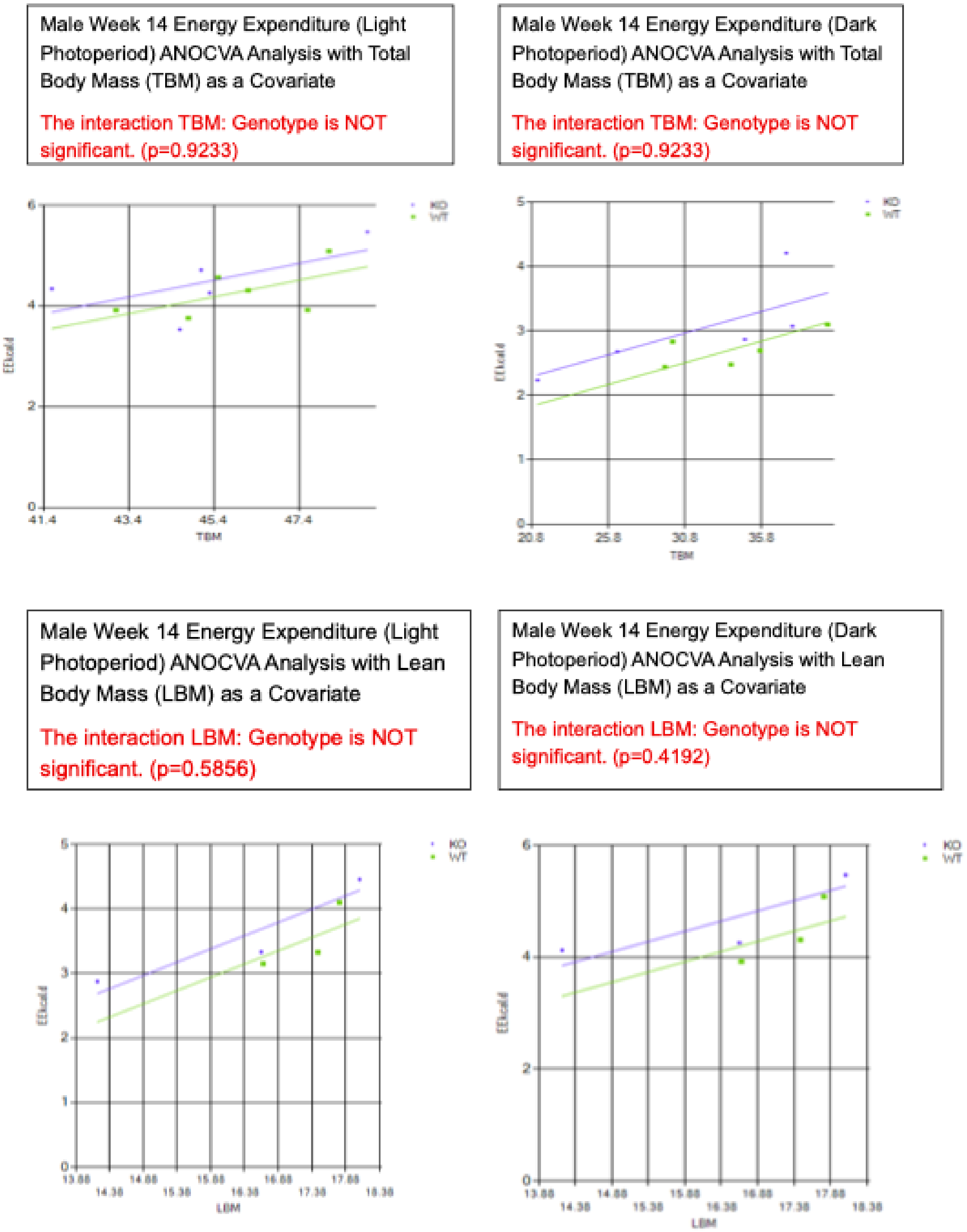

Supplementary Figure 9A: Analysis of energy expenditure (EEkcal.d) of male WT and KO mice by ANCOVA using the total body mass (TBM, upper panels) or lean (fat-free) body mass (LBM, lower panels) during the light and dark cycles.

Female Week 14 Energy Expenditure (Light Photoperiod) ANOCVA Analysis with Total Body Mass (TBM) as a Covariate

The interaction TBM: Genotype is NOT significant. (p=0.5386)

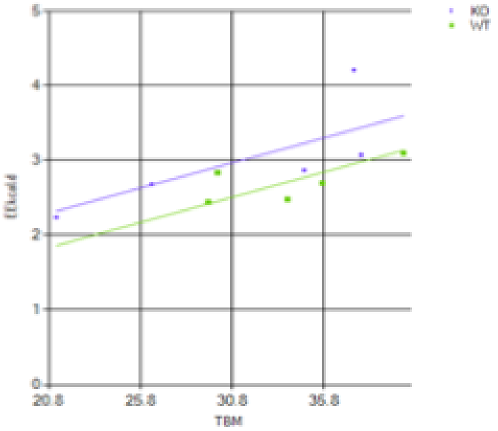

Female Week 14 Energy Expenditure (Dark Photoperiod) ANOCVA Analysis with Total Body Mass (TBM) as a Covariate

The interaction TBM: Genotype is NOT significant. (p=0.4422)

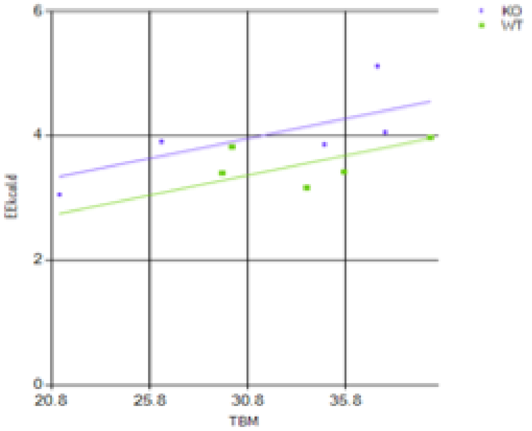

Female Week 14 Energy Expenditure (Light Photoperiod) ANOCVA Analysis with Lean Body Mass (LBM) as a Covariate

The interaction LBM: Genotype is NOT significant. (p=0.5674)

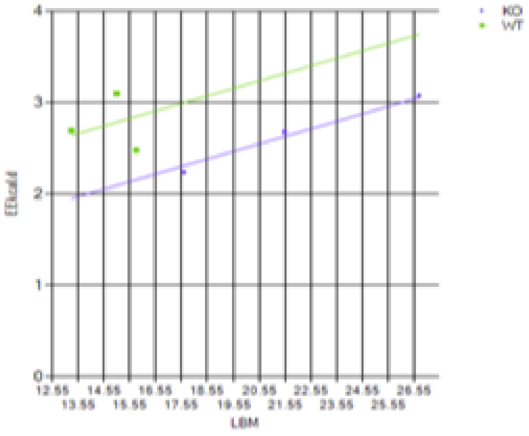

Female Week 14 Energy Expenditure (Dark Photoperiod) ANOCVA Analysis with Lean Body Mass (LBM) as a Covariate

The interaction LBM: Genotype is NOT significant. (p=0.6711)

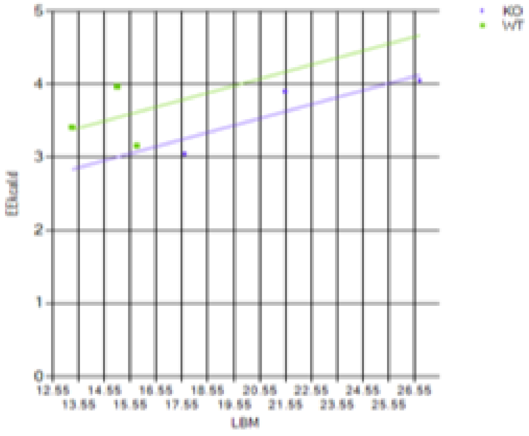

Supplementary Figure 9B: Analysis of energy expenditure (EEkcal.d) of female WT and KO mice by ANCOVA using the total body mass (TBM, upper panels) or lean (fat-free) body mass (LBM, lower panels) during the light and dark cycles.
